# Different syngeneic tumors show distinctive intrinsic tumor-immunity and mechanisms of actions (MOA) of anti-PD-1 treatment

**DOI:** 10.1038/s41598-022-07153-z

**Published:** 2022-02-28

**Authors:** Ying Jin, Xiaoyu An, Binchen Mao, Ruilin Sun, Rajendra Kumari, Xiaobo Chen, Yongli Shan, Mingfa Zang, Ling Xu, Jan Muntel, Kristina Beeler, Roland Bruderer, Lukas Reiter, Sheng Guo, Demin Zhou, Qi-Xiang Li, Xuesong Ouyang

**Affiliations:** 1 Crown Bioscience, Inc, 16550 W. Bernardo Dr. Building 5, San Diego, CA 92127 USA; 2 State Key Laboratory of Natural and Biomimetic Drugs, Peking University, Beijing, 100191 China; 3 Shanghai Model Organisms Center, Inc, Shanghai, China; 4 Biognosys AG, Zurich, Switzerland

**Keywords:** Cancer, Cancer models, Tumour immunology

## Abstract

Cancers are immunologically heterogeneous. A range of immunotherapies target abnormal tumor immunity via different mechanisms of actions (MOAs), particularly various tumor-infiltrate leukocytes (TILs). We modeled loss of function (LOF) in four common anti-PD-1 antibody-responsive syngeneic tumors, MC38, Hepa1-6, CT-26 and EMT-6, by systematical depleting a series of TIL lineages to explore the mechanisms of tumor immunity and treatment. CD8^+^-T-cells, CD4^+^-T-cells, T_reg_, NK cells and macrophages were individually depleted through either direct administration of anti-marker antibodies/reagents or using DTR (diphtheria toxin receptor) knock-in mice, for some syngeneic tumors, where specific subsets were depleted following diphtheria toxin (DT) administration. These LOF experiments revealed distinctive intrinsic tumor immunity and thus different MOAs in their responses to anti-PD-1 antibody among different syngeneic tumors. Specifically, the intrinsic tumor immunity and the associated anti-PD-1 MOA were predominately driven by CD8^+^ cytotoxic TILs (CTL) in all syngeneic tumors, excluding Hepa1-6 where CD4^+^ T_eff_ TILs played a key role. TIL-T_reg_ also played a critical role in supporting tumor growth in all four syngeneic models as well as M_2_-macrophages. Pathway analysis using pharmacodynamic readouts of immuno-genomics and proteomics on MC38 and Hepa1-6 also revealed defined, but distinctive, immune pathways of activation and suppression between the two, closely associated with the efficacy and consistent with TIL-pharmacodynamic readouts. Understanding tumor immune-pathogenesis and treatment MOAs in the different syngeneic animal models, not only assists the selection of the right model for evaluating new immunotherapy of a given MOA, but also can potentially help to understand the potential disease mechanisms and strategize optimal immune-therapies in patients.

## Introduction

Human cancers are not only known as genetic diseases, reflected by the presence of various abnormal genetic alterations within tumor cells, but also manifest as immunological disorders, largely determined by tumor microenvironment (TME), particularly tumor-infiltrate leukocytes (TILs)^1,2^. The aberrant tumor immunity is the foundation of today’s immune-oncology (IO) therapies, including immune checkpoint inhibitors (ICIs)^3,4^, *e.g*., anti-PD-1, anti-PD-L1 or anti-CTLA-4 antibody treatments, which block the intrinsic suppression of tumor immunity. Furthermore, heterogeneous TMEs lead to vastly different responses to ICIs among patients, with only ~ 20% responders in certain sensitive cancer types and the development of resistance among some initial responders. The mechanisms of action (MOAs) underlying these differential ICI sensitivities have yet to be definitively illustrated, although TILs are believed to play significant roles. For example, it has been hypothesized that anti-PD-1 antibody works largely through tumor-reactive CD8^+^ cytotoxic T lymphocytes (CD8^+^-CTLs) within the tumor, as part of TILs, to suppress tumor growth. On the other hand, immunosuppressive functions of tumor-infiltrate regulatory T-cells (T_reg_) or myeloid lineages (*e.g*., M_2_-macrophage and MDSC) have been hypothesized to be critical in suppressing CTLs to enable tumor progression (immune-escape). Some of the immunosuppression can also be inhibited by ICI to activate tumor reactive CTLs^5–7^. However, the contributions of subsets of TILs (non-CD8^+^-CTLs) to ICI-treatments are not well defined, clinically or pre-clinically. Responders may have distinctive anti-tumor MOAs, while non-responders may have different resistant MOAs. Understanding the role or MOAs of different TILs in tumor response to ICI could particularly be meaningful to strategize patient treatment through discovery of novel biomarkers and/or identify effective combination therapies.

Experimental animal modelling ICI pharmacology are valuable in elucidating their underlying MOAs among patient populations^8–10^. Murine syngeneic tumors in immune-competent environment have long been the workhorse for today’s IO research^8,11–14^, not only for proof of concept (POC) but also for investigating the MOAs of novel IO therapeutics. The POC of ICI, *i.e.,* anti-PD-1, anti-PD-L1 and anti-CTLA-4 antibodies, which reverse cancer immunosuppression and promote anti-tumor immune responses in several cancer types, were first demonstrated by using these mouse models^8^. It is also highly plausible to employ them to investigate MOAs of ICI^12,14^, including the roles of different TILs within the TME. A panel of syngeneic tumor models, *e.g*. MC38 murine colon tumor, Hepa 1–6 liver cancer, CT-26 colon cancer and EMT-6 mammary carcinoma, have widely been used for POC or MOA investigations^8,12^. Interestingly, some of them are moderately responsive, while others are resistant to ICIs^12,14^. However, the underlying MOAs of the apparently differential responses remains poorly understood, even for those with similar sensitivity to ICIs. It is prudent to assume that understanding the MOAs in the animal models could have important implications in clinical development of ICIs, guiding patient stratification and combinations.

Two fundamental approaches can be used to investigate disease mechanisms as well as ICI’s MOAs. First, immune-profiling TME baseline along with those upon treatment (pharmacodynamics or PD) can be correlated to pharmacology efficacy and thus can be used to judge the key immune cells or pathways responsible for the diseases or their responses, so called “guilty-by-association”^14^. Second, specific depletion of a given lineage of immune cells or loss of function (LOF) can be also used to delineate the disease mechanisms and ICI MOAs^15^. LOF can be achieved through special methodologies including the administration of the monoclonal antibodies against the lineage-specific markers^16^, or by induced lineage-specific deletion by genetic engineering^17^. Saito et al. described a method to create specific cell ablation in mice to analyze functions of the targeted cell lineages^17^, by conditional cell deletion in transgenic mice (“toxin receptor–mediated cell knockout”). In this system, diphtheria toxin receptor (DTR) was knocked into 3’-UTR of specific lineage marker and administration of diphtheria toxin (DT) caused ablation of the specific cell type. This report describe LOF approaches to understand the critical roles of individual TILs within a given syngeneic model in their impact on tumor growth and ICI efficacy, hence to gain clues on their role in the ICI MOA.

## Results

### Specific immune lineage depletion in a panel of tumor-bearing syngeneic mice

**Figure 1:**
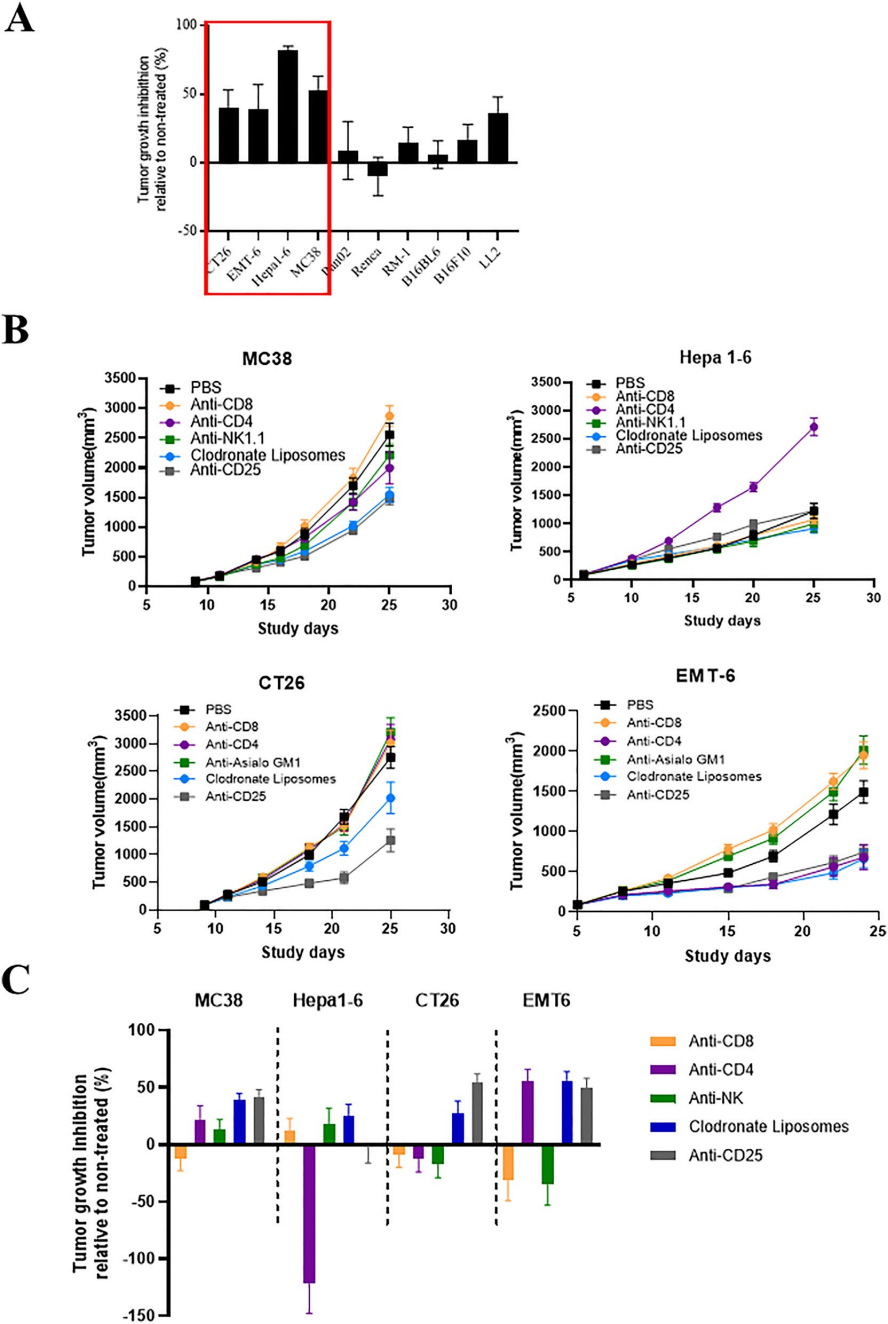
Impact on tumor growth inhibition (TGI) across syngeneic models. (**A**) Response to anti-PD-1 antibody treatment (10 mg/kg) in a panel of syngeneic models (mean % TGI ± SEM) with sensitive models highlighted in red box. (**B**) Growth curves of MC38, Hepa1–6, CT26 and EMT-6 subcutaneous tumors following depletion of specific immune lineages using anti-CD8 (250 mg/mouse), anti-CD-4 (250 mg/mouse), anti-NK (anti-NK1.1 250 mg/mouse for MC38 and Hepa1-6 or anti-asialo for CT26 and EMT-6) and anti-CD25 (400 mg/mouse) antibodies and Clodronate Liposomes (0.2 ml/mouse) with (**C**) TGI summarized in a bar plot (% relative to non-treated control). Individual immune cell profiles for MC38 (**B**) and Hepa1–6 (**C**) assessed by flow cytometry (% of either live or CD45 + cells) following depletion of specific lineages (Experiment 1).

**Figure 2:**
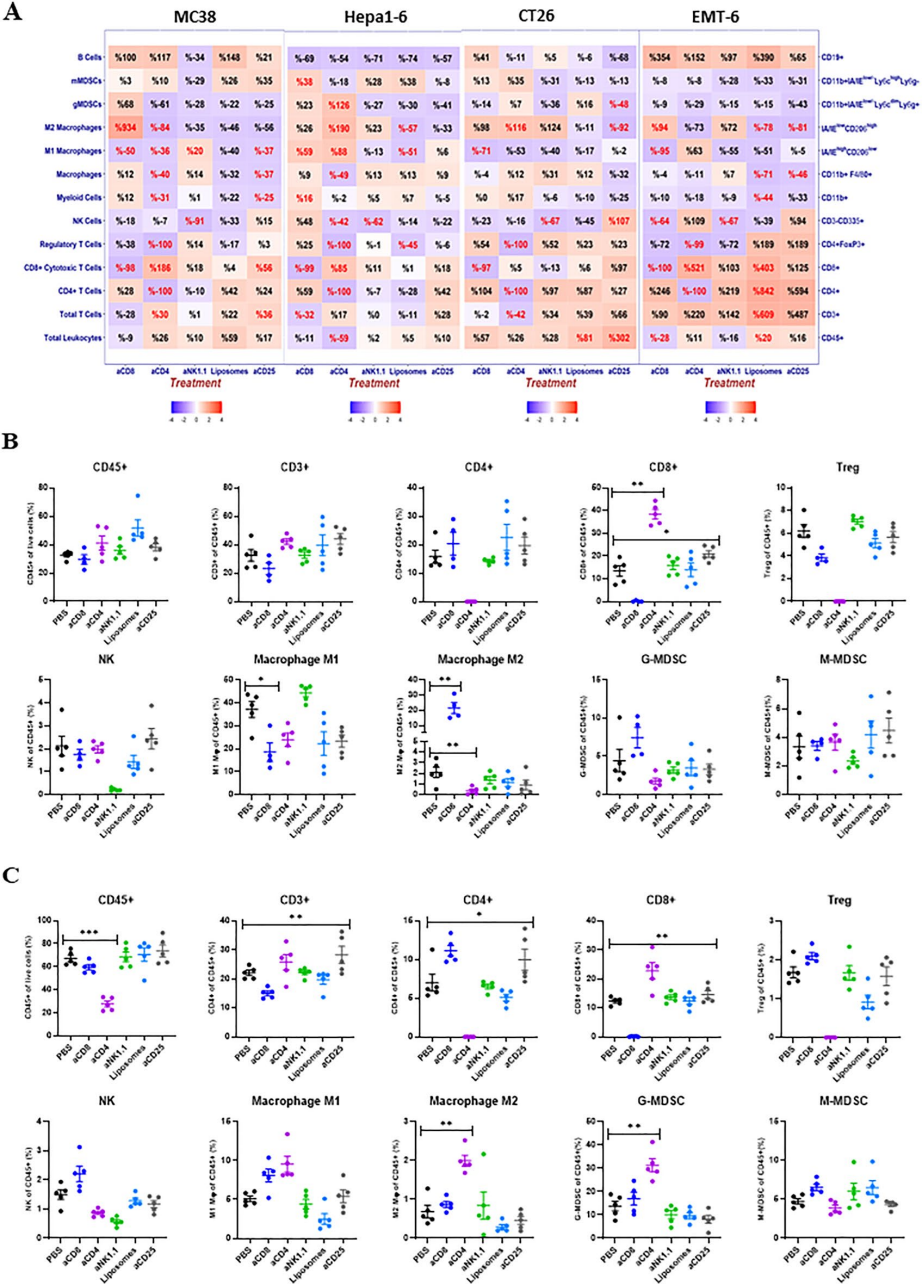
Heat map showing dynamic changes in TILs. (**A)** TIL changes in MC38, Hepa1–6, CT26 and EMT-6 subcutaneous tumors following depletion of specific immune lineages (% TIL relative to non-treated control) represented as either positive or negative values (red or blue shading respectively) with the intensity of color positively-correlating with the absolute value of % change (plotted using R v3.5.2). Text highlighted in red shows significant (p value < 0.05) % changes. Flow cytometry analysis of different immune cells (% of either live or CD45 + cells) of (**B**) MC38 and (**C**) Hepa1-6 tumors following depletion of specific lineages.

LOF by systematic depletion of a specific lineage could be an effective approach to reveal the role of TILs in tumor-bearing syngeneic mice. In the first set of experiments, we systematically depleted syngeneic tumor-bearing mice (MC38, Hepa1-6, CT-26, and EMT-6) of different immune-lineages using the following reagents^16,18^: (1) anti-CD8 (2.43) antibody for removing CD8^+^ CTLs; (2) anti-CD4 (GK1.5) antibody for removing CD4^+^ T-cells; (3) anti-CD25 antibody for CD25^+^ cells that are enriched for T_reg_^18^, (4) either anti-NK1.1 or anti-Asialo-GM1 antibody for removing NK cells, and (5) clodronate liposomes for depletion of macrophage. The selected models (MC38, Hepa1-6, CT26 and EMT-6) are known to be sensitive (or partial) to anti-PD-1 treatment with a tumor growth inhibition (TGI) range of 30–70%, (Table 1) compared to other syngeneic models (Fig. 1A), thus implicating the presence of intrinsic anti-tumor immunity. The impact on tumor growth inhibition (TGI) and changes in the TILs across the 4 syngeneic models following immune cell depletion in comparison to the untreated mice is summarized Table 1 Experiment 1 and Fig. 2A, with representative flow cytometry data for MC38 and Hepa1-6 shown in Fig. 2B,C respectively. Efficient depletion (> 90%) was achieved for CD8^+^ and CD4^+^, and sufficient NK depletion (> 60%) across all 4 syngeneic tumors, as well as the depletion in other tissues (representative depletion data in blood is shown in Supplement Fig. 1C), demonstrating that the approach employed for CD8^+^, CD4^+^ and NK achieved the intended outcome as monitored by multi-color flow cytometry. In contrast, anti-CD25 depletion was transient after single administration (Fig. 2). Anti-Gr-1 antibody for depletion of MDSC was also attempted but not tolerated (data not shown); while clodronate liposomes administration for depletion of macrophage (tumor associated macrophage, or TAM) could not be maintained (likely due to rapid repopulation) (Fig. 2), thus hindering meaningful assessment of the roles of both immune lineages in tumor immunity.

**Table 1:** Summary of MC38, Hepa1–6, CT26 and EMT-6 subcutaneous tumor growth inhibition and % immune cell depletion following treatment for lineage specific depletion either alone (% relative to non-treated control, experiment 1) or in combination with anti-PD-1 antibody treatment (% relative to anti-PD-1 control, experiment 2).

Experiment 1	MC38			Hepa1-6			CT26			EMT6		
Treatment	TGI	% Depletion compared to untreated vehicle		TGI	% Depletion compared to untreated vehicle		TGI	% Depletion compared to untreated vehicle		TGI	% Depletion compared to untreated vehicle	
Anti-PD1	65%	–		58%	–		33%	–		48%	–	
Anti-CD8	− 12%	− 98%		12%	− 99%		− 9%	− 97%		− 31%	− 100%	
Anti-CD4	22%	− 100%		− 122%	− 100%		− 12%	− 100%		55%	− 100%	
Anti-NK	13%	− 91%		18%	− 62%		− 17%	− 67%		− 35%	− 67%	
MØ depletion (clodronate liposomes)	39%	− 32%		25%	13%		27%	12%		56%	− 71%	
Anti-CD25	42%	3%		− 1%	− 6%		54%	23%		50%	189%	

**Experiment 2**	**MC38**			**Hepa1-6**			**CT26**			**EMT6**		
Treatment	TGI compared with vehicle	TGI compared with PD-1	% Depletion compared to anti-PD-1	TGI compared with vehicle	TGI compared with PD-1	% Depletion compared to anti-PD-1	TGI compared with vehicle	TGI compared with PD-1	% Depletion compared to anti-PD-1	TGI compared with vehicle	TGI compared with PD-1	% Depletion compared to anti-PD-1
anti-PD-1	45%			69%			53%			65%		
anti-PD-1/anti-CD8	− 1%	− 83%	− 99%	43%	− 87%	− 98%	− 78%	− 205%	− 95%	− 121%	− 461%	− 93%
Anti-PD-1/anti-CD4	82%	67%	− 99%	− 101%	− 557%	− 100%	46%	− 35%	− 99%	81%	− 147%	− 58%
anti-PD-1/anti-NK	51%	11%	− 70%	47%	− 72%	− 81%	14%	− 46%	− 39%	51%	− 62%	− 2%
Anti-PD-1/MØ-depletion	84%	72%	− 18%	46%	− 76%	64%	76%	54%	3%	75%	29%	− 71%
anti-PD-1/anti-CD25	90%	71%	− 11%	68%	− 6%	100%	94%	82%	− 14%	92%	78%	321%

**Experiment 3**	**MC38**			**Hepa1-6**			**CT26**			**EMT6**		
Treatment	TGI		% Depletion compared to untreated									
DTR-KI-CD8	− 259%		− 98									
DTR-KI-CD4	61%		− 79									
DTR-KI-FoxP3	94%		− 74%									
DTR-KI-NCR1	− 33%		− 53									

**Experiment 4**	**MC38**			**Hepa1-6**			**CT26**			**EMT6**		
				TGI								
DTR-KI-FoxP3				86%								
PD1				82%								
DTR-KI-FoxP3				94%

### The growth kinetics of four syngeneic tumors were differentially impacted by given immune lineage depletions, suggesting distinctive tumor immunity mechanisms

With the confirmation of the depletions of given TIL-lineages, we then monitored the growth kinetics of all four syngeneic tumors with and without depletion (Fig. 1B), with % TGI summarized in Table 1 Experiment 1 and Fig. 1C showing relative changes compared to untreated.

- MC38 tumor: Depletion of CD8^+^ TIL seemed to result in a slight increase in tumor growth (TGI -12%, Table 1 Experiment 1) and NK cell depletion had a slight decrease in tumor growth (TGI 13%). However, these were not significant, only suggesting a possible role of CD8^+^ TIL in controlling tumor growth. In contrast, CD4^+^ depletion, particularly depletion of the CD25^+^ subset (inclusive of T_reg_), seemed to cause a suppression of tumor growth (TGI 22% and 42% respectively), suggesting a potential key role of T_regs_ in promoting tumor growth by suppressing tumor immunity. Similarly, TAM depletion, although transient (32% depletion) appeared to reduce tumor growth (TGI 39%). Taken together this would suggest that immunosuppressive TME mediated via T_regs_ and TAM promoted the growth of MC38 tumor.
- Hepa1-6 tumor.:The depletion of CD8^+^ TILs had little impact on growth kinetics in Hepa1-6 tumor (TGI 12%), while the depletion of CD4^+^ greatly accelerated tumor growth (TGI -122%), suggesting CD4^+^ effector (T_eff_) TILs play a key role in Hepa 1–6 tumor suppression, instead of CD8^+^ CTLs as seen in MC38. CD25^+^ depletion had little effect on tumor growth (TGI -1%), in contrast to MC38 tumor. NK depletion caused a small reduction in tumor growth (TGI 18%) and similarly TAM depletion led to 25% reduction, however stable depletion was not achieved (Table 1 Experiment 1). This data suggests that CD4^+^-T_eff_ cells were key in tumor immunity of Hepa1-6 tumor, an interesting phenomenon less common among syngeneic tumor models.
- CT26 tumor: CD4^+^, CD8^+^ and NK cell depletions slightly promoted tumor growth (TGI − 9%, − 12% and − 17% respectively), however these may be insignificant (Table 1 Experiment 1). In other words, contrasting to MC38/Hepa1-6, the roles of CD4^+^ and CD8^+^ in this tumor remains inconclusive at this stage. On the other hand, CD25^+^ (T_reg_) and TAM depletions seem to cause some degree of tumor growth inhibition (TGI 54% and 27%, respectively), the meaning of which remains elusive due to the lack of stable depletions (23% and 12% depletion respectively, Table 1 Experiment 1).
- EMT6 tumor: CD8^+^ and NK cell depletion both promoted tumor growth (TGI − 31% and − 35%), suggesting CD8^+^-T_CTL_ and NK role in tumor growth inhibition, while CD4^+^, T_reg_ and TAM depletion caused tumor growth inhibition (TGI 55%, 50% and 56% respectively), suggesting their role in an immunosuppression TME (Table 1). Overall, T_CTL_/T_reg_ interaction likely plays major role in tumor immunity in controlling tumor growth.

### Heterogeneous TIL-changes with specific TIL-lineage depletion

Immunophenotype changes (*e.g.* TILs) can also be used for exploring potential mechanism of tumor immunity. Here we examined TIL changes upon the specific lineage depletion treatments in the four syngeneic models.

- TIL-changes associated with CD8^+^-TIL depletion.
  In three syngeneic tumors (MC38, CT26 and EMT6) where TIL-CD8^+^-CTLs seem to play an important role in tumor immunity, CD8^+^-TIL depletion appeared to increase TIL-M_2_-macrophages significantly (934%, 98%, 94% respectively for these three models, Fig. 2A), a TIL subset usually associated with immunosuppression^8^, which correlated with a promotion of tumor growth for these models (− 12%, − 9% and − 31% TGI respectively, Fig. 1C and Table 1 Experiment 1). This result suggests that the dynamics between TIL-CD8^+^ T_CTL_ and M_2_-macrophage determine the inhibition or promotion of tumor growth in these three models. In contrast, for Hepa1-6, where CD8^+^-CTLs seem to play less of a role in tumor immunity described above (Table 1 Experiment 1), the TIL-CD8^+^ depletion did not cause a similar degree of M_2_-macrophage enrichment as in the other three tumors (Fig. 2A).
- TIL-pharmacodynamics of anti-PD-1 treatment associated with CD4^+^-TIL depletion.
  In Hepa1-6 model where TIL-CD4^+^ T_eff_ had an inhibitory effect on tumor growth, CD4^+^-TIL depletion caused dramatic enrichment of TIL-G-MDSC (120%) and TIL-M_2_ macrophages (190%) (both p < 0.01, Figure 2A,C). Since CD4^+^-TIL depletion accelerated the tumor growth, our results suggest that the balance between TIL-CD4^+^-T_eff_ and immuno-suppressive environment inclusive of G-MDSC and M_2_ macrophage play critical roles in the mechanism of this tumor’s immunity (Fig. 2A). This is in contrast to the other three syngeneic tumors where CD8^+^-T_CTL_ TILs are believed to have direct role (*e.g*. MC38, CT26 and EMT6).

### Heterogeneous pharmacological response to anti-PD-1 therapy, influenced by specific lineage depletions, suggesting distinct ICI-MOAs in different syngeneic tumors

**Figure 3:**
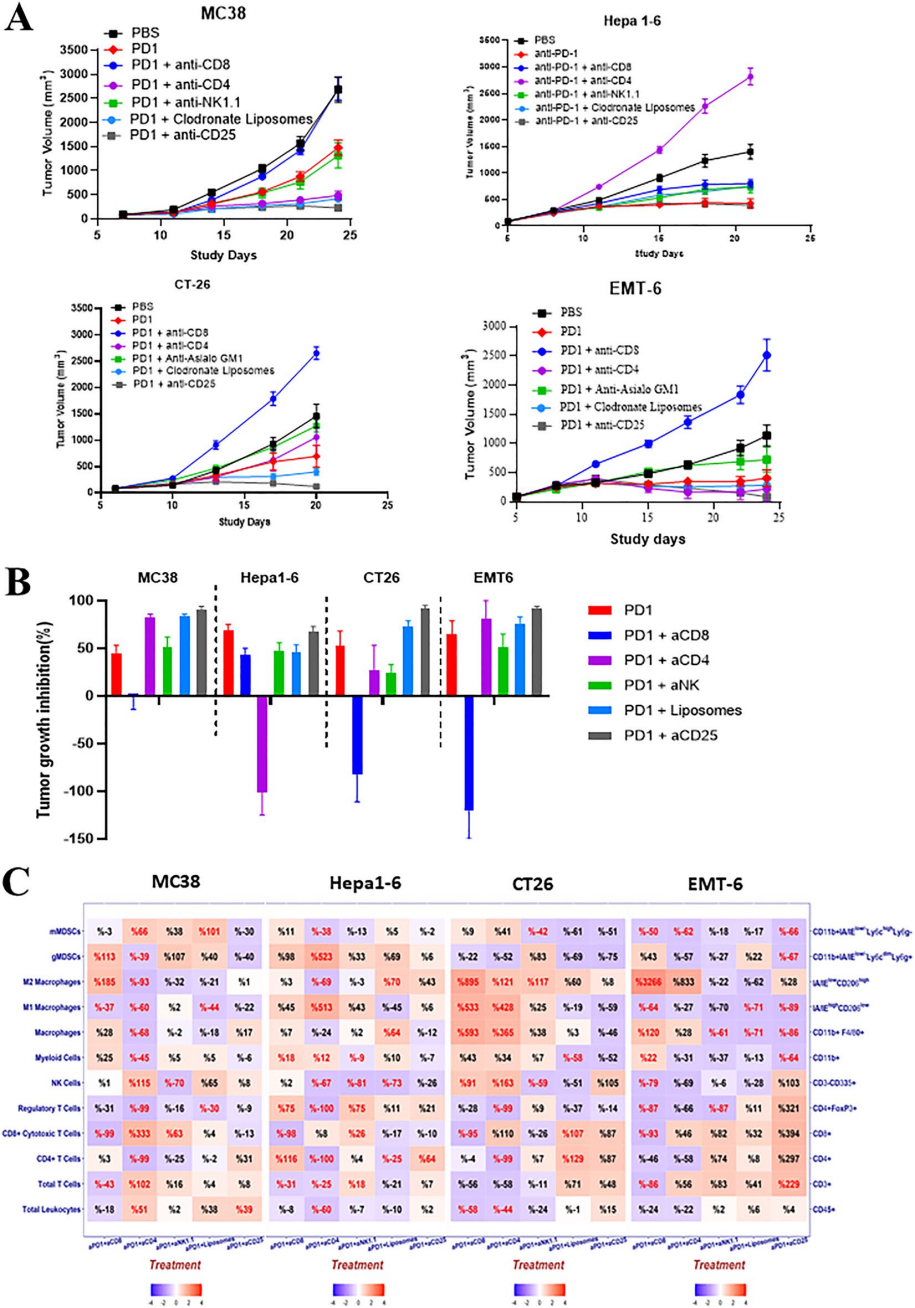
Impact on anti-PD-1 antibody response across syngeneic models (**A**) Growth curves of MC38, Hepa-1–6, CT26 and EMT-6 subcutaneous tumors following depletion of specific immune lineages with anti-PD-1 treatment (10 mg/kg) and (**B**) TGI summarized in a bar plot (% relative to non-treated control). (**C**) Heat map showing dynamic TIL changes in all 4 subcutaneous tumors following depletion of specific immune lineages (% TIL relative to anti-PD-1 control) represented as either positive or negative values (red or blue shading respectively) with the intensity of color positively-correlating with the absolute value of % change (plotted using R v3.5.2). Text highlighted in red shows significant (p value < 0.05) % changes.

With the understanding that different syngeneic tumors have distinct mechanisms of tumor immunity, we hypothesized that ICI-MOA would also be different among different syngeneic models. To test this, we set out to assess the impact of specific lineage-depletion on the responses to anti-PD-1 treatment in the same four syngeneic models, by co-administrations of the above described depletion agents together with anti-PD-1 antibody. The growth kinetics of all four syngeneic tumors upon lineage-depletion, with and without anti-PD-1 treatment were assessed (Fig. 3A), with % TGI summarized in Table 1 Experiment 2 and relative changes compared to untreated control summarized in Fig. 3B.

- MC38 tumor model: While the control tumor mice treated with anti-PD-1 antibody alone demonstrated certain degree of anti-tumor activity (TGI 45%), depletion of CD8^+^ TILs completely abrogated (TGI − 1%) the anti-PD-1 induced tumor inhibition in MC38 (Table 1-Experiment 2 and Fig. 3A,B), suggesting CD8^+^ T_CTL_ were the primary mediator of the anti-PD-1 antibody MOA, consistent with the above observation that T_CTL_ TILs were key in the anti-tumor immunity in MC38 tumors. NK cell depletion had no effect on the anti-PD-1 effect (TGI 51%). Interestingly, CD4^+^ and CD25^+^ cell-depletion as well as TAM depletion (although transient) enhanced the anti-PD-1 antibody effects (82%, 84% and 90% TGI respectively, Table 1 Experiment 2), suggesting that an immune suppressive TME mediated via T_reg_ and TAM counters the anti-PD-1 MOA, whereas in their absence, the anti-PD-1 treatment can be even more effective.
- Hepa1-6 tumor model: Anti-PD-1 antibody alone induced anti-tumor activity (TGI 69%). In sharp contrast to MC38, CD25^+^, NK and CD8^+^ cell depletions had little effect on the anti-PD-1 treatment efficacy (TGI 43%, 47% and 68%, respectively), suggesting a minimal role for NK cells, CD8^+^ T_CTL_- and T_reg_-TILs in the anti-PD-1 treatment MOA in Hepa1-6. CD4^+^ cell-depletion, on the other hand, not only completely abolished the antitumor effect of anti-PD-1-antibody treatment, but also dramatically accelerated tumor growth (TGI -101%, Table 1 Experiment 2 and Fig. 3A,B), suggesting that CD4^+^-T_eff_ mediated the MOA of anti-PD-1 antibody treatment in the Hepa 1–6 tumor, and consistent with above observation that CD4^+^-T_eff_ was the main source of tumor immunity.
- CT-26 tumor model: Anti-PD-1 antibody induced antitumor activity (TGI 53%). CD8^+^ cell-depletion not only completely abolished the efficacy of anti-PD-1 antibody treatment but also dramatically increased tumor growth (TGI − 78%, Table 1 Experiment 2 and Fig. 3B), suggesting T_CTL_-TILs plays vital role in the MOA of anti-PD-1 antibody treatment and tumor immunity. CD4^+^ depletion on the other hand did not affect the anti-PD-1-antibody efficacy (TGI 46%), while CD25^+^  and TAM depletion enhanced anti-PD-1 antibody efficacy (TGI 94% and TGI 76%, respectively), suggesting that, like the MC38 model, an immune suppressive TME mediated via T_reg_ and TAM counters the anti-PD-1 MOA in CT-26 tumor.
- EMT6 tumor model: Anti-PD-1 antibody induced antitumor activity (TGI 65%) and similar to CT26, CD8^+^ cell depletion completely abolished the efficacy of anti-PD-1 antibody treatment as well as accelerated tumor growth (TGI -121%, Table 1 Experiment 2 and Fig. 3B), suggesting T_CTL_-TILs play vital role in the MOA of anti-PD-1 antibody treatment and tumor immunity. CD4^+^ and TAM depletion on the other hand slightly enhanced the anti-PD-1 efficacy (TGI 81% and 75% respectively), while CD25^+^ depletion increased tumor efficacy (TGI 92%), suggesting that the T_reg_ and TAM mediated immune suppressive TME countered the anti-PD-1 MOA. T_reg_ removal improved anti-PD-1 efficacy in this tumor like the MC38 model. Overall, this is also consistent with the above observation that T_CTL_/T_reg_ interaction plays a major role in this model.

Overall, CD8^+^-TILs play a dominant role in mediating the anti-PD-1 MOAs in 3 (MC38, CT26 and EMT-6) of the 4 sensitive models where depletion ablated the sensitivity to ICI. In addition, T_reg_-TILs played an immune suppressive role in these same three models with its depletion resulting in improved ICI response. In contrast, TIL-CD4^+^ T_eff_ play key role in the anti-PD-1 antibody MOA of Hepa1-6 tumor.

### Heterogeneous anti-PD-1 treatment-induced TIL-pharmacodynamics with specific TIL-lineage depletion

Pharmacodynamic effect on immunophenotypes (*e.g.* TILs) can also be used for exploring potential MOAs. We (manuscript in preparation) and others^12,14^ have examined pharmacodynamics of anti-PD-1 treatment in order to elucidate potential ICI treatment MOAs. Here we performed a TIL pharmacodynamic analysis of anti-PD-1 antibody treatment following different TIL lineage depletion in the four syngeneic models. The % immune cell depletion in relation to anti-PD-1 treatment is summarized in Table 1 Experiment 2 and dynamic changes are shown in Fig. 3C.

- TIL-pharmacodynamics of anti-PD-1 treatment associated with CD8^+^-TIL depletion.
  In three syngeneic tumors (MC38, CT26 and EMT6) where CD8^+^-CTL-TILs seem to be directly responsible for anti-PD-1 mediated tumor inhibition, CD8^+^-TIL depletion combined with anti-PD-1 treatment appeared to increase TIL-M_2_-macrophage (185%, 895%, 3266% respectively for these three models) (Fig. 3C), a TIL subset usually associated with immunosuppression^8^, which correlated with a promotion of tumor growth for these models (− 1%, − 78% and − 121% TGI respectively, Fig. 1C and Table 1 Experiment 2). This result suggests that the dynamics between TIL-CD8^+^ T_CTL_ and M_2_-macrophage determine the inhibition or promotion of tumor growth in these three models, which is largely in agreement with the observations of increasing CD8^+^-TILs by anti-PD-1 treatment (manuscript in preparation) and depletion of CD8^+^ cells abrogating the efficacy. In contrast, for Hepa1-6, where CD8^+^-CTL seems to play less role in anti-PD-1 response as described above (Table 1 Experiment 2), the TIL-CD8^+^ depletion together with anti-PD-1 treatment did not cause M_2_-macrophage enrichment in the tumors (Fig. 3C), but instead resulted in an increase in the levels of CD4^+^ T cells (116%) and T_reg_ (75%) potentially suggesting an equilibrium between TIL-T_reg_ and TIL-CD4^+^ T_eff_ in Hepa1-6 model, correlating to (rendering) the observed tumor growth inhibition by anti-PD-1 treatment through CD4^+^-T_eff_ and T_reg_. In another word, TIL-CD8^+^ T-cells play little role in anti-PD-1 treatment MOA in Hepa 1-6 model.
- TIL-pharmacodynamics of anti-PD-1 treatment associated with CD4^+^-TIL depletion.
  In Hepa1-6 model where TIL-CD4^+^ T_eff_ has an inhibitory effect on tumor growth, CD4^+^-TIL depletion and anti-PD-1 treatment caused dramatic enrichment of TIL-G-MDSC (>500%, Fig. 3C), another TIL subset usually associated with the suppression of tumor immunity^8,19,20^. Since CD4^+^-TIL depletion with anti-PD-1 treatment completely lost the efficacy in Hepa1-6, our results suggest the balance between TIL-CD4^+^-T_eff_ and immuno-suppressive environment inclusive of G-MDSC playing critical roles of anti-PD-1 MOAs in this tumor. This is in contrast to the other three syngeneic tumors where CD8^+^-T_CTL_ TILs are believed to have direct role (*e.g*. MC38, CT26 and EMT6), with TIL-CD4^+^ depletion inducing different degree of TIL-CD8^+^ T_CTL_ increases, particularly in MC38 where an increase of 333% was observed (Fig. 3C) which also resulted in an increase in TGI (84%, Fig. 3 and Table 1). However, from Fig. 3C, the overall TIL-pharmacodynamics profiles are different among these three models, therefore contributing to the anti-PD-1 efficacy in different ways which are still to be explored.

### Heterogeneous anti-PD-1 treatment immunogenomic-pharmacodynamics with the given TIL-lineage depletion

**Figure 4:**
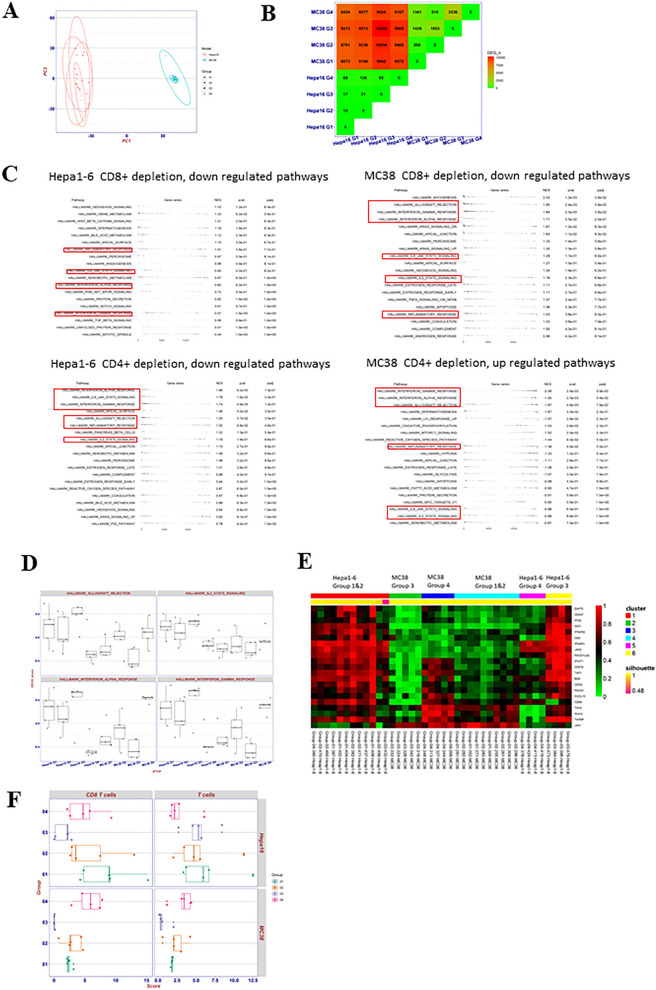
Transcriptome analysis of Hepa1-6 and MC38 tumors from different treatment groups (Group 1 PBS; Group 2 anti-PD-1; Group 3 anti-PD-1 treatment plus CD8 + depletion and Group 4 anti-PD-1 treatment plus CD4 + depletion). (**A**) PCA of differential expressed genes (DEGs); (**B**): DEG analysis; (**C**) Table plot for selected pathways down/up regulated with immune related pathways highlighted in red box; (**D**) pathway activity score by GSVA; (**E**) Heat map of consensus cluster analysis for 21 immunity related genes; (**F**) mMCP-counter to assess CD8^+^ T cells and total T cell populations.

In addition to TIL-pharmacodynamics, we sought to explore treatment MOAs at molecular levels through immunogenomic analysis of treated tumor samples. First, we performed global transcriptomics analysis (RNAseq) on different treatment groups (PBS, anti-PD-1 treatment, anti-PD-1 plus CD8^+^ depletion and anti-PD-1 plus CD4^+^ depletion) of the two selected syngeneic models, MC38 and Hepa1-6 tumors, for their contrasting tumor immunity. For this transcriptome pharmacodynamic analysis, we employed several statistical methods, attempting to identify potential involved genes and pathways that could be part of the MOAs. Principle component analysis (PCA)^21^ was first performed, revealing that treatment groups of specific models clustered together (Fig. 4A), which implicated that transcriptomic differences between mouse syngeneic models dominated transcriptomic differences between treatment groups of the same syngeneic model.

We then performed differential expressed genes (DEGs) analysis^21^ followed by gene set enrichment analysis between treatment groups of Hepa1-6 and MC38 models, we found the number of DEGs between the two models are much higher (> 8000) than the number of DEGs between different treatment groups of the same model (< 4000, Fig. 4B), consistent with the above PCA. We also found that CD8^+^ depletion/anti-PD-1 treatment caused mild decrease (insignificant) of anti-tumor immunity for Hepa1-6 (normalized enrichment score (NES) < 1, Fig. 4C), while severe decrease of anti-tumor immunity for MC38 (NES > 1.5, p < 0.05 compared to anti-PD-1 treatment) per downregulation of major immune response gene pathways (Fig. 3C). In comparison, CD4^+^ depletion/anti-PD-1 treatment resulted in severe decrease of anti-tumor immunity for Hepa1-6 (NES > 1.5, p < 0.05) while a strong increase of anti-tumor immunity for MC38 (NES < − 1.5, p < 0.05) per immune-response pathways down-regulation in Hepa1-6 and up-regulation in MC38 tumors (Fig. 4C). We calculated pathway activity score by gene set variation analysis (GSVA)^22^, and we observed that anti-PD-1 treatment increased interferon gamma (IFN-γ) response in MC38, which was further boosted by CD4^+^ depletion (p value 0.011 compared to PBS Group 1 and 0.0011 compared to Group 3 CD4^+^ depletion/anti-PD-1 treatment, Fig. 3D), but was reversed by CD8^+^ depletion, which was consistent with the IFN-γ NES (− 2.04 p value 0.0024 for CD4^+^ depletion and 1.71 for CD8^+^ depletion, Fig. 4C). In Hepa1-6 CD4^+^ depletion caused severe decrease of IFN-γ response upon anti-PD-1 treatment (p value 0.0232 compared to PBS Group 1 and 0.0187 compared to Group 3 CD4^+^ depletion/anti-PD-1 treatment, Fig. 4D) which also correlated with the IFN- γ NES score (1.74, p value 0.0049, Fig. 4C). These pathway changes are highly correlated to the observed anti-tumor pharmacology of these models (Fig. 3B and Table 1 experiment 2) and the TIL-pharmacodynamic (Fig. 3C) data above.

We next conducted cluster analysis based on 21 critical immunity related genes to determine the silhouette score^23^, which demonstrates how similar an object is to its own cluster compared to other clusters, ranging from − 1 to + 1. Six clusters were identified with an average silhouette score of 0.97 suggesting a high degree of cohesion within clusters (Fig. 4E). Immune cell depletion treatment-naïve Hepa1-6 tumor (cluster 1) displayed a higher score in comparison to MC38 (cluster 4) across the majority of the immunity related genes suggesting a “immunogenically hotter” phenotype than that of MC38. Treatment with anti-PD-1 did not influence the scoring in either model. For Hepa1-6, CD4^+^ depletion with anti-PD-1 treatment severely repressed the expression of the majority of those critical immunity related genes (cluster 5, Fig. 4E), while CD8^+^ depletion upon anti-PD-1 treatment had minimal effect other than inhibiting CD8A expression (cluster 6). For MC38, CD4^+^ depletion upon anti-PD-1 treatment enhanced the expression of many immunity-related genes such as CD8αA IFN-γ and the genes related to antigen presentation machine (B2M, HLA-A, TAP1, TAP2 and TAPBP) (cluster 3, Fig. 4E), while CD8^+^ depletion upon anti-PD-1 treatment further repressed the expression of those immunity related genes (cluster 2, Fig. 4E). All these are consistent with TIL-pharmacodynamic data and efficacy data above.

We used murine Microenvironment Cell Population (mMCP)-counter^24^ to estimate the immune composition from transcriptomic data. For Hepa1-6 model, CD8^+^ depletion upon anti-PD-1 treatment resulted in a decrease in CD8^+^ TILs, but the overall T cell population was comparable with both vehicle group and anti-PD-1 treated group (Fig. 4F and Supplemental Fig. 2). On the other hand, CD4^+^ depletion upon the treatment resulted in a decrease of overall T cell population (Fig. 4F), although statistically insignificant. In MC38 model, CD8^+^ depletion upon the treatment caused a significant decrease of CD8^+^ TILs (p value 0.0012, Fig. 4F) and overall small decrease in T-TILs. However, CD4^+^ depletion upon treatment resulted in a significant increase of CD8^+^ TIL (p value 0.0037, Fig. 4F) and small increase in total T-TILs. All the mMCP-counter studies are consistent with our above TIL pharmacodynamic results which is consistent with our MOA hypothesis per the above studies: CD4^+^-TILs (T_reg_) plays key role in suppression of anti-tumor immunity (CD8^+^ T_CTL_) in MC38 tumors and CD4^+^-T_eff_ TILs plays key role in anti-tumor immunity in Hepa1-6 tumors.

### Heterogeneous anti-PD-1 treatment proteomic pharmacodynamics with given TIL-lineage deletion

**Figure 5:**
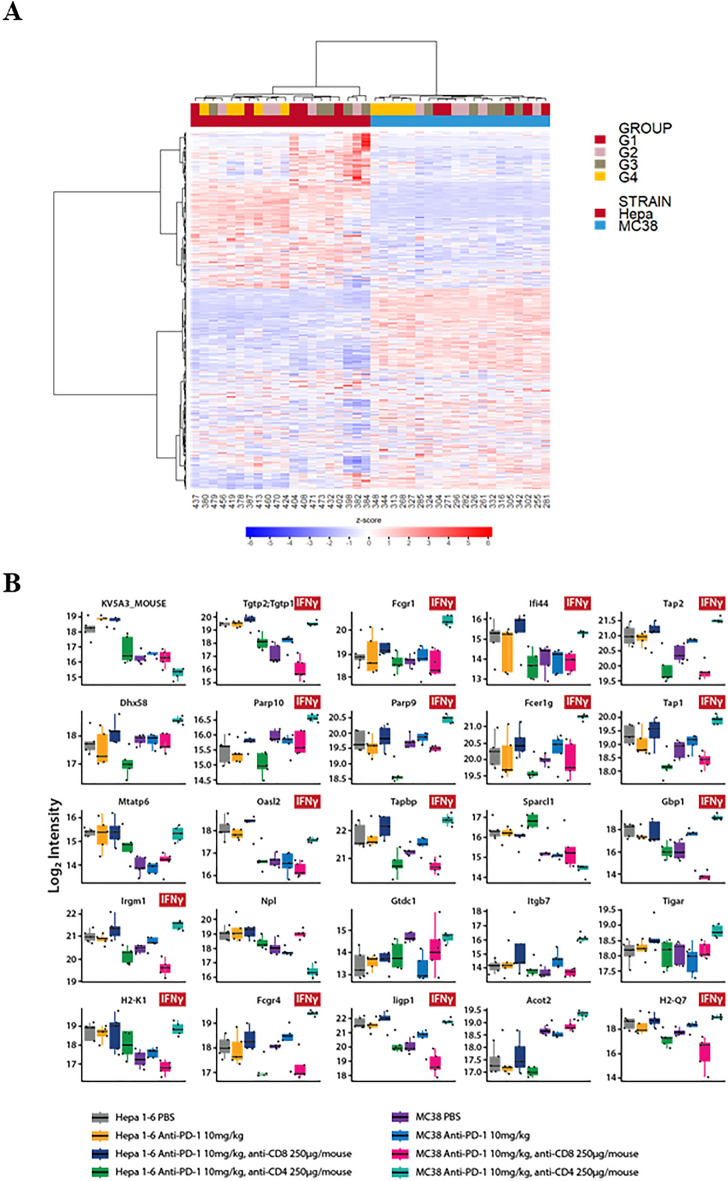
Deep proteomics analysis of MC38 and Hepa1-6 tumors under different treatments. (**A**) Unsupervised hierarchical clustering analysis represented as a heatmap (z-score transformed data, distance metric: Manhattan Distance; linkage strategy: Ward’s Method) and (**B**) Functional analysis of the top 25 IFNγ signalling markers identified from partial least squares discriminant analysis (PLS-DA) of tumors from Group 1 PBS; Group 2 anti-PD-1 treatment; Group 3 anti-PD-1 treatment plus CD8 + depletion and Group 4 anti-PD-1 treatment plus CD4 + depletion.

Multi-omics are increasingly recognized to be even more powerful tools in mechanistic and biomarker discovery studies, as compared to single-omics. With that, we attempted proteomics readouts of pharmacodynamic analysis, in addition to the transcriptomic readouts, so additional expressing-omic perspective can be provided. To this end, we next explored the treatment impact on immune-pathways at the whole proteomic levels upon depletion of CD8^+^ and CD4^+^ TILs in MC38 and Hepa1-6 tumors. We performed global proteomic analysis (> 9000 proteins) on tumor samples from different treatment groups in order to reveal whether they can be categorized into different classes with biological meaning. Figure 5A shows the heatmap results of the unsupervised hierarchical clustering analysis. Differences in protein levels between the two types of tumors in different mouse strains could be clearly distinguished, as anticipated and consistent with that based on genomics above (Fig. 4A). Only MC38 tumors treated with anti-CD4 and anti-PD-1 antibodies (Group 4, Fig. 5A) were categorized together but other treatment groups were not.

Detailed proteomic analysis revealed distinctive changes in immune pathways between MC38 and Hepa1-6 tumors upon the depletion of CD8^+^ and CD4^+^ TILs (Table 2, Supplemental Fig. 3). First, in MC38 tumor, proteins in MHC class I antigen presentation pathways and cytotoxicity were enhanced upon anti-PD-1 treatment, and the additional CD4^+^-TIL depletion further boosted these pathway changes (Table 2 and Supplemental Fig. 3), suggesting that anti-PD-1 caused tumor death due to increased anti-tumor immunity, which is boosted by the CD4^+^ depletion (CD4^+^ suppresses anti-tumor activity in MC38, consistent with hypothesis MOA per above observations). However, in Hepa1-6 model, contrary effects were observed (Supplemental Fig. 2, Table 2) where CD4^+^ depletion caused decrease of anti-tumor immunity and tumor death, confirming CD4^+^ TILs’ direct role in anti-tumor activity, also consistent with early hypothesized the T_eff_ MOA on Hepa1-6; second, for the combo of anti-PD-1 and anti-CD4^+^ depletion group, immune suppression markers (*e.g.* PDL-1,2, LAG3, SHP2, etc.) increased in MC38 tumor model, while decreased in Hepa1-6 model, which could potentially explained by a feedback of tumor to the treatment (Supplemental Fig. 2 and Table 2); third, strong upregulation of IFN-γ inducible proteins upon anti-PD-1 therapy was observed in MC38 model, which is boosted upon CD4^+^ TIL depletion, while many IFN-γ pathway related markers were downregulated in the combo group of anti-PD-1 and anti-CD4 in Hepa1- 6 (Fig. 5B, 12 out of the 25 top candidate proteins identified from the PLS-DA analysis are involved in IFN-γ signaling cascade). These observations were consistent with the observed pharmacology efficacy and our MOA hypothesis per above studies: CD4^+^-TILs (T_reg_) plays key role in suppression of anti-tumor immunity (CD8^+^ CTLs) in MC38 tumors and CD4^+^-TILs (T_eff_) plays key role in anti-tumor immunity in Hepa1-6 tumors.

**Table 2:** Summary of immune pathway dynamics upon treatment.

	Treatment	Hepa1-6			MC38		
		Anti-PD-1	Anti-PD-1 with CD8^+^ depletion	Anti-PD-1 with CD4^+^ depletion	Anti-PD-1	Anti-PD-1 with CD8^+^ depletion	Anti-PD-1 with CD4^+^ depletion
	Efficacy	Yes	Yes	No	Yes	No	Yes
Pathways	Immuno-suppression	–	–	↓	↑	↓	↑
	Immuno-activation	–	–	↓	↑	↓	↑
	Ag-presentations (MHC-I)	–	–	↓	↑	↓	↑
	Cytotoxicity	–	–	↓	↑	↓	↑
	IFN-γ	–	–	↓	↑	↓	↑

↑ upregulation or ↓ downregulation of markers.

### Specific immune lineage depletion by DTR-engineered mice largely confirmed heterogeneous tumor immunity and anti-PD-1 MOAs

In recognition of certain limitations of lineage depletion by administration of antagonist agents (pharmacology approach), *e.g*. toxicologic and pharmacokinetic limitations, genetic engineering approaches to deliver induced systemic and stable lineage depletion was also employed. We created a series of immune-lineage-specific DTR-mice in C57BL/6 background: CD8a-IRES-DTR-EGFP, CD4-IRES-DTR-EGFP, FoxP3-IRES-DTR-EGFP, Ncr1-IRES-DTR-EGFP for deleting CD8^+^ T-, CD4^+^ T-, T_reg_, NK-cells, respectively, in order to further confirm our observations above by pharmacology methods. These knock-in (KI) mice were engineered to express DTR-EGFP downstream of specific lineage markers, *i.e.* expression of DTR/EGFP were co-regulated with endogenous lineage markers without disrupting endogenous expression. We first set out to test two syngeneic tumors with the most contrasting mechanisms, MC38 and Hepa1-6.

**Figure 6:**
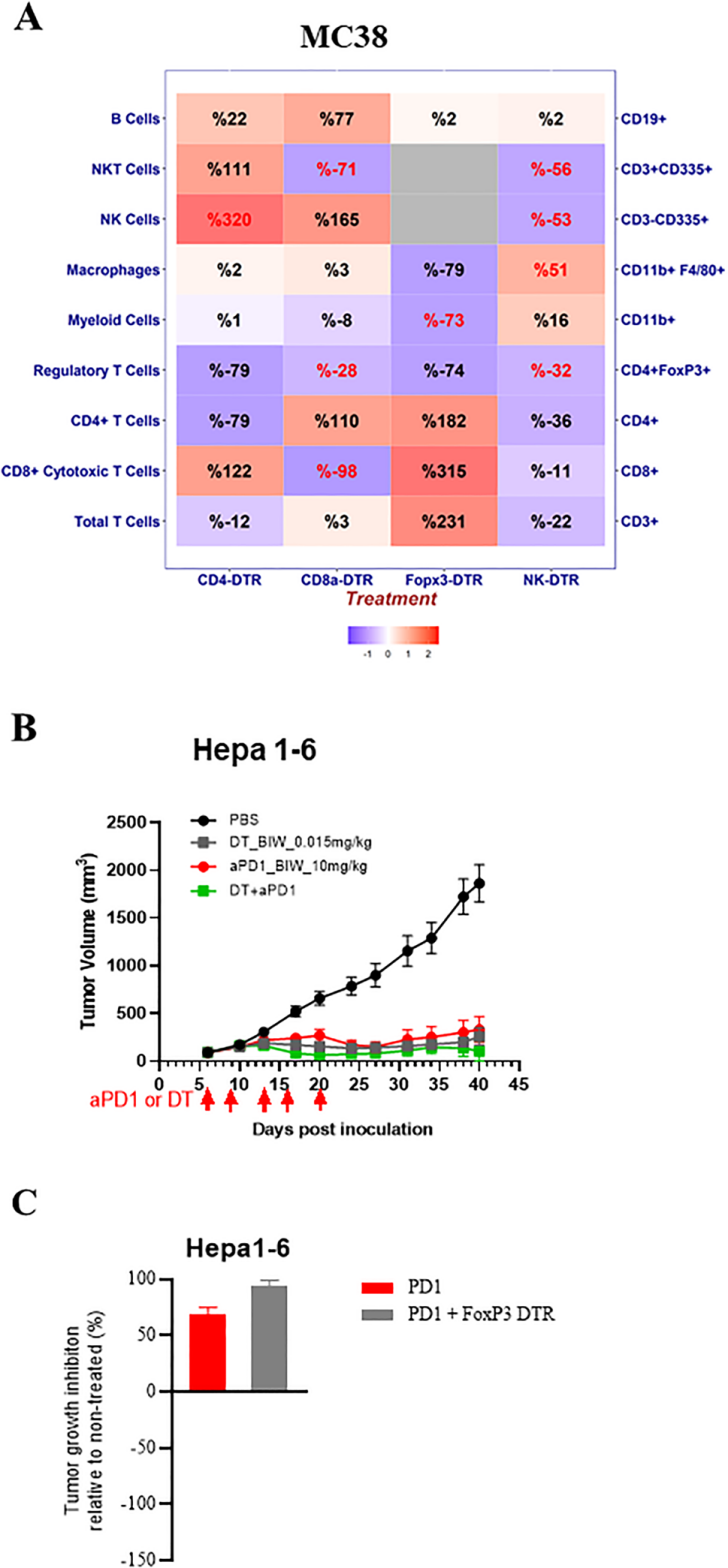
Impact of targeted immune cell depletion in DTR transgenic mice; (**A**) Heat map of TIL pharmacodynamic assessed by FACS (% TIL relative to untreated control) in DTR mice bearing MC38 tumors following specific immune cell depletion, represented as either positive or negative values (red or blue shading respectively) with the intensity of color positively correlating with the absolute value of % change (plotted using R v3.5.2). Text highlighted in red shows significant (p value < 0.05) % changes. (**B**) Growth curve of Hepa-1–6 subcutaneous tumors following treatment of Foxp3-DTR mice with DT with and without anti-PD-1 treatment (10 mg/kg) with (**C**) TGI summarized in a bar plot (% relative to non-treated control).

- MC38 tumor. MC38 tumors were engrafted into these 4 strains of DTR-knock-in (DTR-KI) mice along with the wild-type (naïve) mice, followed by the DT treatment, and monitored for the depletion of specific immune cell lineages over time in blood, spleen, and TILs. As anticipated, the DT treatment effectively depleted CD8^+^ T-cells, CD4^+^ T-cells, T_reg_ and NK cells in peripheral blood in the respective DTR-KI mice, (Supplemental Fig. 4), along with spleen (Supplemental Fig. 5) and tumors at the terminations (Fig. 6A and Supplemental Fig. 5). We then monitored the growth kinetics of MC38 syngeneic tumor in these mice, as compared to their growth in the naïve mice (and Supplemental Fig. 6A–D with TGIs summarized in Table 1 Experiment 3). CD8^+^-depletion accelerated tumor growth, suggesting an anti-tumor role for CD8^+^ TIL on MC38 tumor model (Supplemental Fig. 6A). Both CD4^+^ cell and T_reg_ (a subset of CD4^+^ with FoxP3^+^, also a subset of CD25^+^ with FoxP3^+^) depletion inhibited tumor growth (Supplemental Fig. 6B,C respectively), with T_reg_ depletion inducing almost complete inhibition, suggesting that T_reg_ plays important role in promoting MC38 tumor growth, consistent with the response observed with anti-CD4 and anti-CD25 antibody above (Fig. 3C). NK depletion had little impact on tumor growth (Supplemental Fig. 6D) also consistent with anti-NK depletion study.
- Hepa1-6. As shown earlier, the impact of the antibody mediated depletions of CD4^+^ and CD25^+^ (a subset of CD4^+^ and inclusive of T_reg_) were quite distinct in the impact of Hepa1-6 growth, contrasting to the more consistent influence on MC38 tumors, making the role of T_reg_ in Hepa1-6 inconclusive. To further investigate the effects of T_reg_ depletion in Hepa1-6 tumors, we performed a head-to-head comparison of Hepa1-6 tumors engrafted in either Foxp3-DTR mice or naïve C57BL/6 mice, treated with repeated doses of DT or single dose of CD25 antibody respectively, with or without anti-PD-1 treatment. In the Foxp3-DTR mice, we observed Hepa1-6 tumor growth inhibition with DT treatment (Table 1 experiment 4 and Fig. 6B,C), suggesting that T_reg_ actually have similar immune-suppression effect as in MC38. However, as shown in Table 1, anti-CD25 antibody treatment did not result in tumor growth inhibition (Fig. 1B), nor improve the anti-PD-1 efficacy (Fig. 3A) in Hepa 1–6 tumors, which likely resulted from that CD25 antibody targets CD4^+^ effector cells as well as T_reg_, a T_reg_ specific response could not be delineated with this method. The DTR-KI line therefore had an advantage in robust depletion of a desired lineage without impacting other immune cell populations.

## Discussion

The baseline and/or PD profiling TILs has been a key methodology to investigate MOAs of a given immunotherapy. However, this methodology is largely built on the “correlative” relation, not “causal-effect”, so less definitive. MOA investigation via syngeneic tumor modelling with high intrinsic experimental variations, particularly in the case of immunotherapies, often render immune-profiling-only approaches less reliable/conclusive, although still commonly used^8,12,14^ (and our own unpublished studies). Alternative methodology is to knock-out certain tumor immunity (“KO”) or LOF^15^, which can potentially be more conclusive, as compared to the “association”-based approaches. Our data using LOF methodology across a panel of four syngeneic tumor models (partially) sensitive to anti-PD-1 antibody treatment clearly demonstrated the presence of distinctive intrinsic tumor immunities in each model and distinct MOAs of ICI-treatment, reflected largely by their unique TIL components which likely resulted in the underlying molecular mechanisms (immunological pathways). Our work based on LOF largely confirms some of the observations in the studies based on tumor immunophenotyping^12,14^ (our unpublished data). It is also highly plausible that different mechanisms reported here could be mapped to patient populations, where similar LOF study cannot be performed. In another word, this type of preclinical-only interrogation may have significant clinical value. If so, this type of “KO” or LOF methodology using animal models would be particularly powerful for investigating IO MOA, even beyond ICI treatments.

Due to being the most important components of tumor immunity, LOF by depleting specific TIL subsets should be a reasonable approach to interrogate mechanisms of disease and treatments. However, the effectiveness of the experimental approaches for LOF may vary. For instance, administration of agents targeting specific markers can be generally satisfactory for many cases, *e.g.* monoclonal antibodies targeting CD4, CD8, CD25, etc., but may have limitations in some other cases, including incomplete depletion, insufficient pharmacokinetics (repopulation dynamics), toxicity and non-surface markers inaccessible by antibody etc*.*, rendering those efforts futile or less productive, *e.g*. macrophage (incomplete depletion due to repopulation), MDSC (lethal), FoxP3 (inaccessible by antibody), etc. On the other hand, genetic engineered induced lineage depletion seems to provide clearer results in certain cases, although much more difficult to establish. Nevertheless, our limited data (MC38 and Hepa 1–6 tumor only) confirms the observations in the studies using targeting agents: different subtypes of T-TILs and myeloid lineages played important role in tumor immunity and ICI MoA, consistent with many as previously suggested^12,14^ (our unpublished data), whereas NK cells played a minimal role. With these being said, combining the two approaches to achieve LOF can in some cases be more revealing than single approach. One such example was the T_reg_ depletion: the CD25 antibody mediated depletion slowed down MC38 tumor growth, but not Hepa1-6 tumor growth; in addition it enhanced anti-PD-1 efficacy in several tumor types but not Hepa1-6; however, Foxp3^+^ T_reg_ depletion by engineering/DT treatment enhanced inhibition of Hepa1-6 tumor growth, specifically and persistently, which was explained by the possibility that CD25 antibody also targeted CD4 T_eff_ that were required for Hepa1-6 tumor growth, thus providing additional insight into the tumor immunity.

Each syngeneic tumor is unique, in terms of either tumor cells or TME, which is provided by host, but ultimately determined by the tumor genetics in a defined environment. It is worth emphasizing that, due to the remarkable consistence between existing tumor immunity (*e.g.* TILs) and anti-PD-1 treatment MOAs we observed among the four models tested, it seems that the pre-existing tumor immunity (baseline) dictates the MOA of a given I/O therapy and its efficacy. In another word, the unique TILs in a given tumor is likely to dictate tumor immune-pathogenesis and thus MOAs of IO therapies, or could be used as predictive biomarker for IO therapy. To this end, different syngeneic models may have very different utilities in assessing I/O therapies, or selection of models is vital to the success of IO pharmacology evaluation of any given novel IO treatments. It is thus prudent to continue to this line of experimentation to address the tumor immunity and I/O MOAs for other syngeneic models beyond the four models described in this report. It would be ideal ultimately that all the common syngeneic models are being classified according to their unique tumor immunity, so to be optimally utilized. Furthermore, it will be particularly useful to investigate other ICIs in the different syngeneic models in the context of distinctive tumor immunity. For example, it would be meaningful to look at anti-CTLA-4 antibody, since its MOA is still somewhat under debate, whether efficacy has been suggested to be attributed to intrinsic CD8^+^ T cell activation by T_reg_ depletion via ADCC or down-regulation^16^.

One thing remains unclear is whether the distinctive tumor immunity we observed has similar counterparts in human tumors, so that the I/O treatment MOAs would also be similar and the knowledge gained in the preclinical modeling can be effectively applied in the clinic. For instance, the T_CTL_/T_reg_ interactions in controlling tumor growth in MC38 tumor would be similarly observed in certain patients and that the MOA of I/O for those patients would be also similar^16^. If it holds true in the clinic that intrinsic tumor immunity mechanism (say TILs) determines the MOAs of a given IO therapy as our studies suggested, understanding individual patient’s existing tumor immunity mechanisms, as potentially revealed by immunophenotyping, immunogenomics and immune proteomics will no doubt become a very useful approach to guide I/O treatments in patients. Practical biomarkers that can accurately reveal intrinsic tumor immunity would likely to be vital to the future I/O therapy.

## Materials and methods

### Animals

C57BL/6 J and Balb/c mice (6–8 weeks’ old) were purchased from Shanghai Lingchang Biotechnology Co., Ltd (China). The series of immune lineage-DTR (diphtheria toxin receptor) knock-in transgenic mouse models, CD8a-IRES-DTR-EGFP, CD4-IRES-DTR-EGFP, Ncr1-IRES-DTR-EGFP and Foxp3-IRES-DTR-EGFP, were created, by knocking in an expression cassette containing internal ribosome entry site (IRES), human diphtheria toxin receptor (DTR), and enhanced green fluorescent protein (EGFP), downstream of the internal stop codon of the corresponding gene (*e.g*. forkhead box P3 (Foxp3) gene) as shown in Supplemental Figs. 7–10. We have previously shown that by *i.p*. dosing of 0.015 mg/kg DT twice per week to the MC38 tumor bearing Foxp3DTR mice, Foxp3^+^ T_reg_ can be eliminated in blood, spleen and TIL. General animal procedures have been widely described previously^25,26^. Mice were housed under specific pathogen-free conditions in Polysulfone IVC cage and supplied with irradiated standard rodent chow and 0.2 μm filtered, autoclaved reverse osmosis water ad libitum. All the protocols and amendment(s) or procedures involving the care and use of animals were reviewed and approved by the Crown Bioscience Institutional Animal Care and Use Committee (IACUC) prior to conducting the studies. The care and use of animals were conducted in accordance with AAALAC (Association for Assessment and Accreditation of Laboratory Animal Care) International guidelines as reported in the Guide for the Care and Use of Laboratory Animals, National Research Council (2011). All animal experimental procedures were under sterile conditions at SPF (specific pathogen-free) facilities and conducted in strict accordance with the Guide for the Care and Use of Laboratory Animals from The National Institutes of Health, AVMA guidelines for Euthanasia of Animals (2020) and ARRIVE guidelines^27^.

### Syngeneic tumor model

The murine liver cancer cell line Hepa1-6 and colon cancer cell line CT26 were purchased from Shanghai Institutes for Biological Sciences (China), breast cancer cell line EMT-6 was purchased from ATCC. MC38 was purchased from FDCC (FuDan IBS Cell Center). Tumor cells were cultured in DMEM (Gibco) or RPMI1640 (Gibco) with 10% FBS (Excell) at 37ºC in an atmosphere of 5% CO_2_ in air. Cells in exponential growth phase were harvested and re-suspended in PBS for animal inoculation. Implantation procedures for transplanted tumor in mice have been previously widely described^15,26^. Briefly, Balb/c mice were injected subcutaneously with 5 × 10^5^ cells for either CT26 and EMT-6, or C57BL/6 J or DTR mice were injected subcutaneously with either 1 × 10^6^ cells MC38 or 5 × 10^6^ cells Hepa1-6 cells. Routine body weight and tumor size were measured twice a week until endpoint and tumor volume was determined as length × width^2^ × 0.5.

### In vivo antibody depletion and treatment

Five to nine days post inoculation, when the tumors were well established (mean tumor volume was approximately 80-100mm^3^), the mice were randomized based on tumor volume into treatment and vehicle control groups and treatment initiated. Each group comprising 10–16 mice for wildtype mice, n = 8 for Foxp3-DTR mice with Hepa1-6 tumors and n = 2 for DTR mice with 2–8 wild type C57BL/6 mice matched as control for MC38 studies.

Anti-mouse PD-1 antibody (RMP1-14; BioXcell) was dosed i.p. at 10 mg/kg twice a week for up to 3 weeks either alone or in combination (Experiment 2). CD8, CD4 T cells and NK cells (in C57BL/6) were depleted in vivo by i.p. injection of 250 μg/mouse of 2.43, GK1.5, or PK136 mAb (BioXCell) respectively, twice a week for 2 to 3 weeks. For T_reg_ depletion, mice were injected i.p. with a single dose of 400 μg/mouse of anti-CD25 mAb (clone PC-61.5.3, BioXCell) 4 days before tumor inoculation^16^. For macrophage depletion, mice were injected i.v. with 0.2 mL liposomal clodronate (FormuMax) twice a week for 2 to 3 weeks. For NK cell depletion in Balb/c mice, an i.p. injection of anti-Asialo-GM-1 (clone Poly21460, Biolegend) was given at dose level of 20 μl/mouse once every 5 days for up to 3 weeks.

DTR mice were treated with diphtheria toxin (DT, Sigma-Aldrich) at 0.15 mg/kg i.p. every other day for 3 doses^28^, except for Foxp3-DTR line which was dosed twice weekly (5 doses in total), either alone or in combination with anti-PD-1.

Tumor growth inhibition (TGI) was calculated as TGI % = 1—ΔT/ΔC where T and C are the mean tumor volume of the treated and control groups, respectively. An in vivo TGI > 70% was referred to as sensitive to treatment, whereas TGI < 40% was not and TGI 40–70% was referred to as partial responsive with progressive disease. The tumor, blood and spleen samples were collected 2 to 3 days post the last dose of anti-PD-1 antibody and analyzed by flow cytometry, RNAseq and proteomic analysis.

### Flow analysis

Phenotyping TIL and leukocytes in non-tumor organs by multi-color flow cytometry have been previously described^13,15^. Antibodies used are summarized in Supplemental Tables 1 and 2, with list of markers and gating strategy summarized in Supplemental Fig. 1.

### Transcriptomics

The tumor samples from experiment 2 (MC38 and Hepa1-6) were collected 2 to 3 days post the last dose of anti-PD-1 antibody and RNA sequencing carried out as previously described^21^. R package (version 3.5.2) DESeq2^29,43^ was used to identify differentially expressed genes and to perform principle component (PC) analysis. First, we removed low expressed genes if they were not expressed in more than 80% samples. Then we used R package surrogate variable analysis (sva)^30^ to remove batch effects.

Differential expressed genes (DEGs) were determined by an absolute value of log2 fold change higher or equal to 1 and adjusted p-value less than 0.05^21^.

We used R package fgsea^31^ to conduct gene set enrichment analysis, based on hallmark gene sets of MSigDB^32,33^. Enriched gene sets were ranked according to their differential expression significance and fold change (NES), with the most significantly regulated genes at the top of table plot. We also used R package GSVA to conduct gene set variation analysis^22^ and assessed significance between any two specific groups for each pathway using Benjamini–Hochberg method.

R package CancerSubtypes^34^ was used to conduct cluster analysis based on 21 key immunity related genes, which were T-cell receptor genes (CD3G, CD4, CD8A, ZAP70 and CD247), antigen presentation machinery genes (B2M, HLA-A, TAP1, TAP2, TAPBP), interferon gamma pathway genes (IFNG, IFNGR1, IFNGR2, JAK1, JAK2, STAT1 and CXCL10) and immune checkpoint blockade genes (PDCD1, PDCD1LG2, IDO1 and CD274). Consensus clustering method^35^ was used to identify cancer subtypes, and the highest silhouette score could be obtained when the number of cluster was set to 6.

R package mMCP-counter^24^ was used to estimate mouse immune and stroma cell populations from bulk tissue RNA-Seq data. First transcripts per million (TPM) normalization was done for RNA-Seq data, then 12 immune and 4 stromal murine cell populations were estimated based on highly specific transcriptomic markers. Statistical significance was determined by pairwise comparison using t tests with non-pooled SD.

### Sample preparation for proteomics

FFPE samples (5 µm curls) were prepared after an adapted protocol from Buczak et al.^36,37^. In brief, FFPE curls were twice deparaffinized using Xylenes and afterwards rehydrated using a series of different ethanol/water solutions (100% (v/v), 90%, 70%) followed by an incubation step in 0.1% (v/v) formic acid/water. The supernatant was removed and lysis buffer (0.1 M Dithiothreitol (DTT), 4% (w/v) Sodium dodecyl sulfate (SDS), 0.1 M Tris–HCl pH10) was added to the samples. Samples were lyzed by sonication in the Bioruptor (Diagenode, Seraing, Belgium) with following settings: 15 cycles, 60 s ON, 30 s OFF, 4 °C, high intensity. Afterwards the samples were incubated for 1 h, at 99 °C and 500 rpm. The sonication was repeated followed by a second boiling step. After cooling of the sample to room temperature the samples were alkylated by addition of iodoacetamide (IAA) to a final concentration of 0.05 M and incubation for 30 min in the dark. For clean-up of the lysate, the samples were subjected to acetone precipitation and acetone pellets were digest by subsequent incubation with Lysyl Endopeptidase (LysC, 3 h, 37C, 1000 rpm, Wako Chemicals, Richmond, VA) and trypsin (o/n, 37C, 1000 rpm, Promega, Madison, WI). The digestion reaction was stopped by addition of Trifluoroacetic acid (TFA). Peptides were purified using 96-Well BioPureSPE Midi clean-up plates (NEST group, Southborough, MA) following manufacturers protocol. Eluates were dried completely in a speed-vac (Savant SPD131DDA, Thermo Fisher Scientific, San Jose, CA). The samples were resuspended in buffer A (1% acetonitrile, 0.1% formic acid in water) containing iRT peptides (Biognosys, Schlieren, Switzerland). Peptide concentration were determined using nano-drop (Spectrostar Nano, BMG labtech, Ortenberg, Germany). Concentrations were adjusted to 1 µg/µl.

### LC–MS analysis for library generation (data-dependent acquisition, DDA)

For library generation, a pool of all samples was prepared (200 µg). The pool was subjected to high pH HPRP fractionation using a Dionex Ultimate 3000 LC (Thermo Scientific, Sunnyvale, CA) and an ACQUITY UPLC CSH1.7 µm C18 column (2.1 × 150 mm, Waters, Milford, MA). Peptides were separated by a 30 min non-linear gradient from 1% HPRP buffer B (100% acetonitrile) / 99% HPRP buffer A (20 mM ammonium formate, pH 10) to 40% buffer B. A micro fraction was taken every 45 s and pooled into 12 final fractions. Pooled fractions were dried completely in a speed-vac and resuspended in buffer A containing iRT peptides. Peptide concentrations were determined using nano-drop.

Fractionated samples (1ug) were separated by a non-linear gradient from 5% buffer B (80% acetonitrile, 0.1% formic acid in water) / 95% buffer A (0.1% formic acid in water) to 59% buffer B in 96 min on an in-house packed 60 cm column (PicoFrit emitters, ID75um, New Objective, Woburn, MA; CSH 1.7um column material, Waters, Milford, MA) using an Easy nLC 1200 (Thermo Fisher Scientific, San Jose, CA) coupled online to a Q Exactive HF-X mass spectrometer (Thermo Fisher Scientific). Flow rate was set to 250 nl/min. The Q Exactive HF-X mass spectrometer was operated in data-dependent Top15 mode with following settings: resolution: MS1 scan range: 350–1650 Th; 60,000; MS1 AGC target: 3e6; MS1 maximum injection time (IT): 25 ms; MS2 scan resolution: 15,000; MS2 AGC target: 2e5; MS2 maximum IT: 25 ms; isolation window: 4 Th; scan range: 200 to 2000 Th; NCE: 27; minimum AGC target: 1e3; only charge states 2 to 6 considered; peptide match: preferred; exclude isotopes: on; dynamic exclusion: 14 s.

### LC–MS analysis for quantification (data-independent acquisition, DIA)

The DIA acquisition method was adapted from Bruderer et al.^38^. Peptides (2 µg) were separated by a non-linear 192 min gradient from 2% buffer B to 59% buffer B using the same LC–MS setup as described before. The mass spectrometer was operated in DIA mode using the following settings for the MS1 scan: range: 350 to 1650 Th, resolution: 120,000; AGC target: 3e6; maximum IT: 20 ms.The MS1 scan was followed by 35 DIA scan using the following settings: resolution: 60,000; AGC target: 3e6; maximum IT: 118 ms; fixed first mass: 250 Th; stepped NCE: 25.5, 27, 30. The window widths were adjusted to precursor density and overlapped by 0.5 Th.

### Data analysis—Hybrid library generation

The DDA and DIA raw files were searched separately with SpectroMine 2.0.190613.43665 (Biognosys) against the mouse UniProt FASTA (downloaded on Jul 1st, 2019) using the following settings: fixed modification: carbamidomethyl (C); variable modifications: acetyl (protein N-term), oxidation (M), methylation (KR); enzyme: trypsin/P with up to two missed cleavages. Mass tolerances were automatically determined by SpectroMine and other settings were set to default. Search results were filtered by a 1% FDR on precursor, peptide and protein level^39,40^. For hybrid library^41^ generation, the search archives from the above mentioned were used for library generation in SpectroMine. Default settings were applied during library generation (1% FDR).

### DIA data analysis

Prior analysis of the DIA data, the raw files were converted into htrms files using the htrms converter (Biognosys). MS1 and MS2 data were centroided during conversion. The other parameters were set to default. The htrms files were analyzed with Spectronaut 13 (version: 13.5.190812, Biognosys) using the previously generated hybrid library and default settings^42^. The results were filtered by a 1% FDR on precursor and protein level (Q value < 0.01).

## Supplementary Information

Supplementary Information.
