# Supplementary material for: Different syngeneic tumors show distinctive intrinsic tumor-immunity and mechanisms of actions (MOA) of anti-PD-1 treatment
Source: Sci Rep. 2022 Feb 28;12:3278. doi: 10.1038/s41598-022-07153-z (PMC8885837; doi:10.1038/s41598-022-07153-z)
Supplement: Supplementary file 1 — Supplementary Information. [file 41598_2022_7153_MOESM1_ESM.pdf]

Supplemental Table 1: List of antibodies for flow analysis of specific markers for antibody/reagent depletion studies

Markers	Fluorochrome	Clone	Cat.	isotype	Vender
**CD45**	BV785	30-F11	103149	Rat IgG2b， κ	Biolegend
**CD3**	BUV395	17A2	740268	Rat IgG2b， κ	BD
**CD4**	BV421	GK1.5	100438	Rat IgG2b， κ	Biolegend
**CD8**	PE-eFluor610	53-6.7	61-0081-82	Rat IgG2a， κ	eBiosciences
**Foxp3**	PE	FJK-16s	12-5773-82	Rat IgG2a， κ	eBiosciences
**CD335**	BV605	29A1.4	137619	Rat IgG2a, κ	Biolegend
**CD11b**	PE-Cy7	M1/70	101216	Rat IgG2b, κ	Biolegend
**F4/80**	BV510	BM8	123135	Rat IgG2a， κ	Biolegend
**I-A/I-E**	AF700	M5/114.15.2	107622	Rat IgG2b, κ	Biolegend
**CD206**	APC	C068C2	141708	Rat IgG2a， κ	Biolegend
**Ly-6G**	BUV737	1A8	741813	Rat IgG2a， κ	BD
**Ly-6C**	FITC	HK1.4	128006	Rat IgG2c， κ	Biolegend
**PD-1**	BV650	J43	744546	Armenian Hamster IgG_2, κ	BD
**PD-L1**	Percp-cy5.5	10F.9G2	124334	Rat IgG2b, κ	Biolegend
**CD19**	BV711	6D5	115555	Rat IgG2a， κ	Biolegend
**L/D**	efluo780	NA	65-0865-14	NA	eBiosciences

Supplemental Table 2: List of antibodies for flow analysis of specific markers for DTR depletion studies

Markers	Fluorochrome	Clone	Cat.	isotype	Vender
**CD45**	BUV661	30-F11	565079	Rat IgG2b， κ	BD
**CD3**	BUV395	17A2	740268	Rat IgG2b， κ	BD
**CD4**	BV421	GK1.5	100438	Rat IgG2b， κ	Biolegend
**CD8**	PE-eFluor610	53-6.7	61-0081-82	Rat IgG2a， κ	eBiosciences
**Foxp3**	PE	FJK-16s	12-5773-82	Rat IgG2a， κ	eBiosciences
**CD335**	BV605	29A1.4	137619	Rat IgG2a, κ	Biolegend
**CD11b**	PE-Cy7	M1/70	101216	Rat IgG2b, κ	Biolegend
**F4/80**	BV510	BM8	123135	Rat IgG2a， κ	Biolegend
**I-A/I-E**	AF700	M5/114.15.2	107622	Rat IgG2b, κ	Biolegend
**CD206**	Percp-cy5/5	C068C2	141716	Rat IgG2a， κ	Biolegend
**Ly-6G**	BV785	1A8	127645	Rat IgG2a, κ	Biolegend
**Ly-6C**	APC	HK1.4	128016	Rat IgG2c， κ	Biolegend
**CD19**	BV711	6D5	115555	Rat IgG2a， κ	Biolegend
**EGFP**	FITC				
**L/D**	efluo780	-	65-0865-14	-	eBiosciences

A.

Marker	immune cell Population
CD45	Total leukocytes
CD3	Total T cells
CD4	CD4+ T helper cells
CD8	CD8+ Cytotoxic T cells
CD4+FoxP3+	Regulatory T cells
CD11b+IA/IE$^{low/-}$Ly6c^{high}Ly6g-	M-MDSC
CD11b+IA/IE$^{low/-}$Ly6c^{dim}Ly6g+	G-MDSC
CD11b+ F4/80	Macrophages
IA/IE^{high}CD206^{low}	M1 Macrophages
IA/IE^{low}CD206^{high}	M2 Macrophages
CD3-CD335+	NK cells
CD3+CD335+	NKT cells
CD19	B cells
Live/Dead (fixable)	Live/Dead

B.

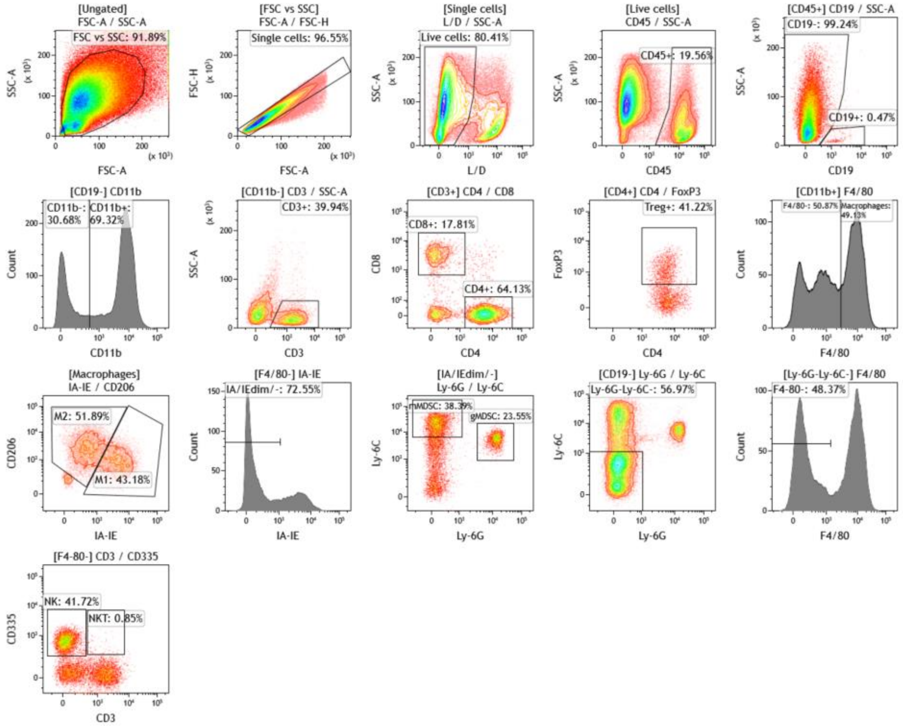

C

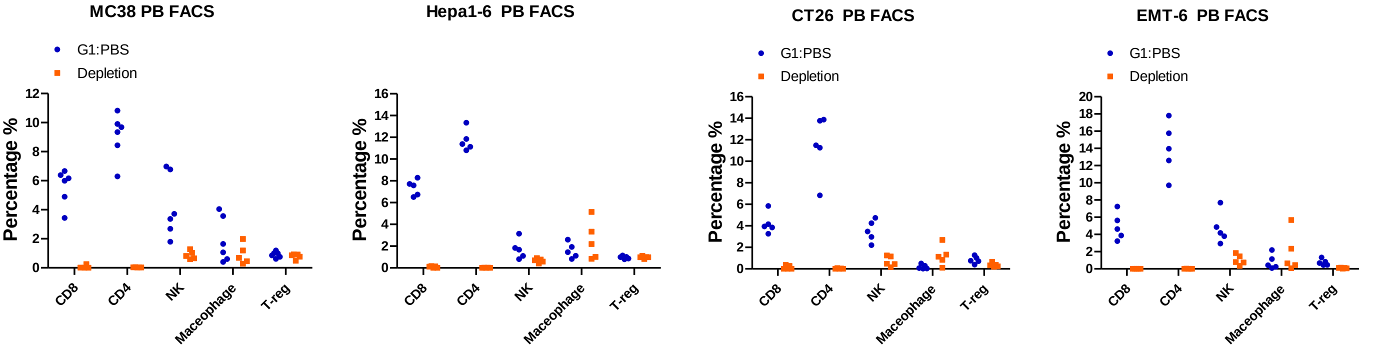

Supplement Figure 1. Overview of flow analysis A) list of FACs markers for immune cell analysis; B) gating strategy used and C) Changes in immune cells in peripheral blood from mice bearing MC38, Hepa1-6, CT-26 and EMT-6 tumors (% of either live or CD45+ cells) following depletion of specific lineages using anti-CD8 (250 μg/mouse), anti-CD-4 (250 μg/mouse), anti-NK (anti-NK1.1 250 μg/mouse) and anti-CD25 (400 μg/mouse) antibodies and Clodronate Liposomes (0.2 ml/mouse) without anti-PD-1 treatment (Experiment 1).

Hepa 1-6

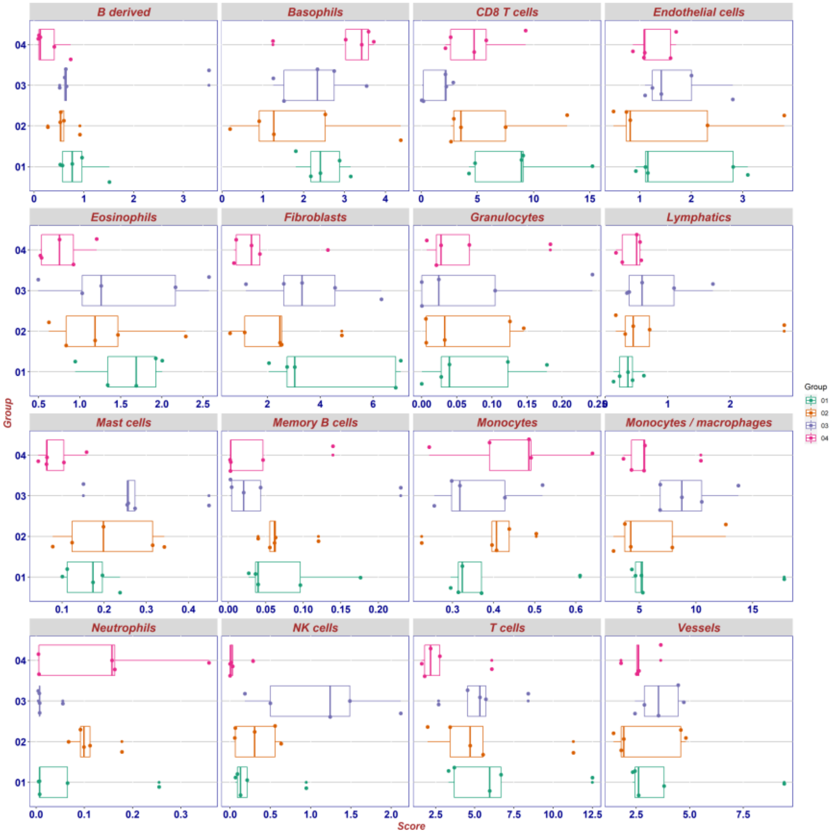

MC38

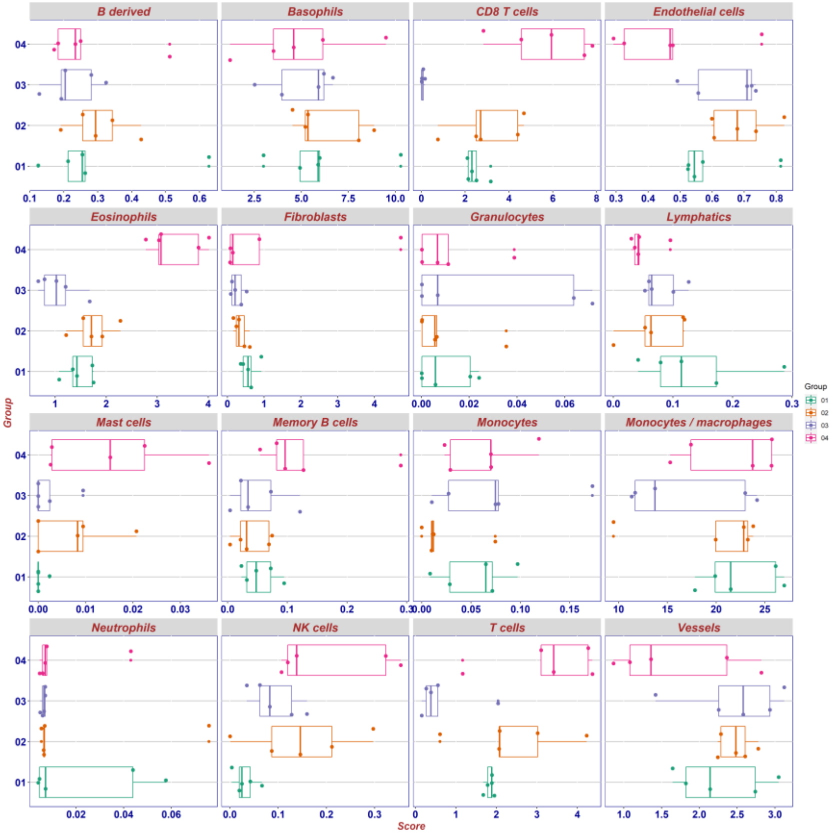

Supplemental Figure 2. A murine Microenvironment Cell Population (mMCPcp)-counter to assess CD8 T cells and T cell populations of Hepa1-6 and MC38 tumors from different treatment groups (Group 1 PBS; Group 2 anti-PD-1; Group 3 anti-PD-1 treatment plus CD8+ depletion and Group 4 anti-PD-1 treatment plus CD4+ depletion).

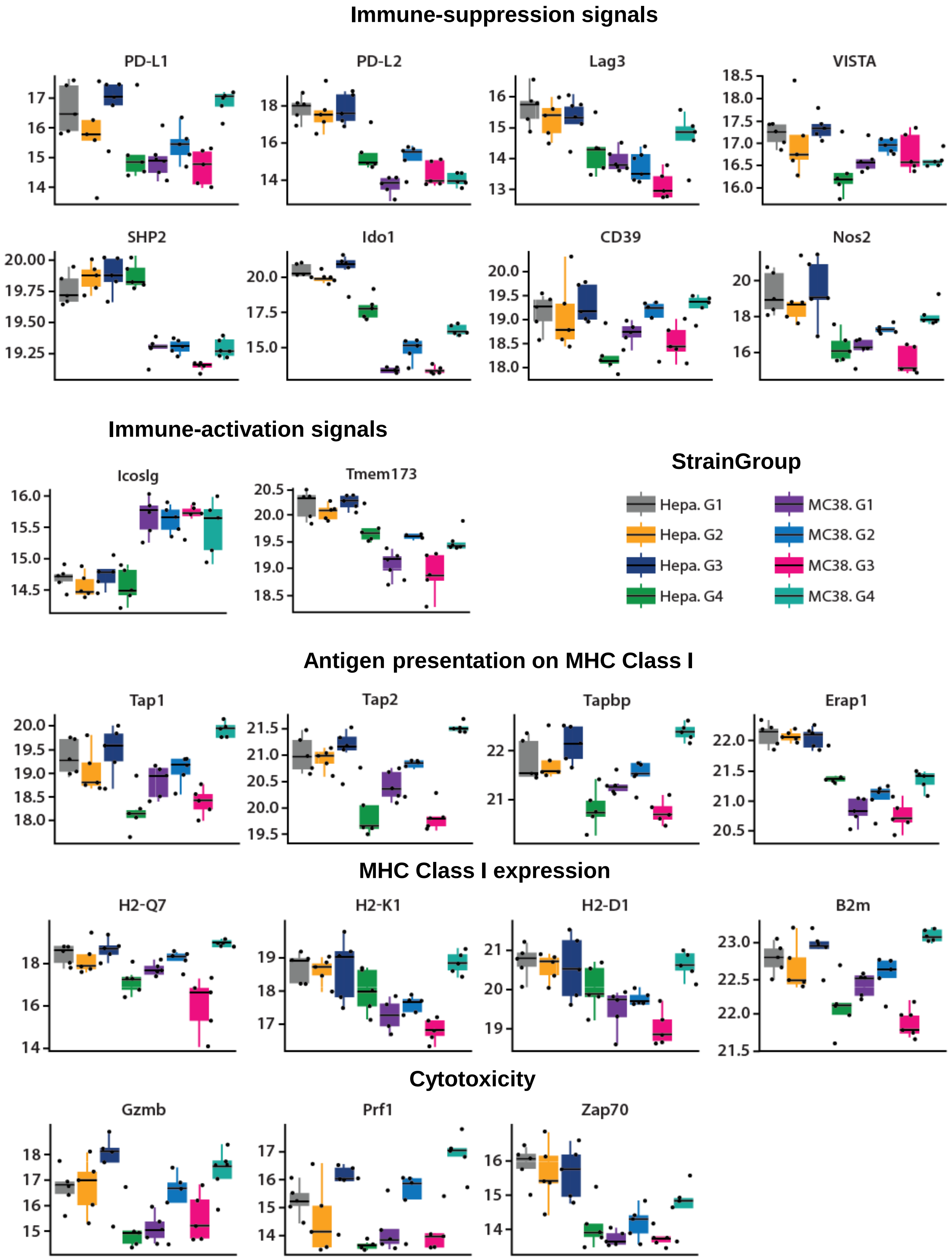

Supplement Figure 3. Effects of CD4$^+$ depletion on antigen presentation, cytotoxicity pathways and immune modulating signals. Functional analysis of the top candidate markers identified from partial least squares discriminant analysis (PLS-DA) for Group 1 PBS; Group 2 anti-PD-1 treatment; Group 3 anti-PD-1 treatment plus CD8+ depletion and Group 4 anti-PD-1 treatment plus CD4+ depletion from both Hepa 1-6 and MC38 tumors. CD4$^+$ TIL depletion leads to upregulation of MHC I proteins, improved antigen presentations & boosted cytotoxicity in MC38

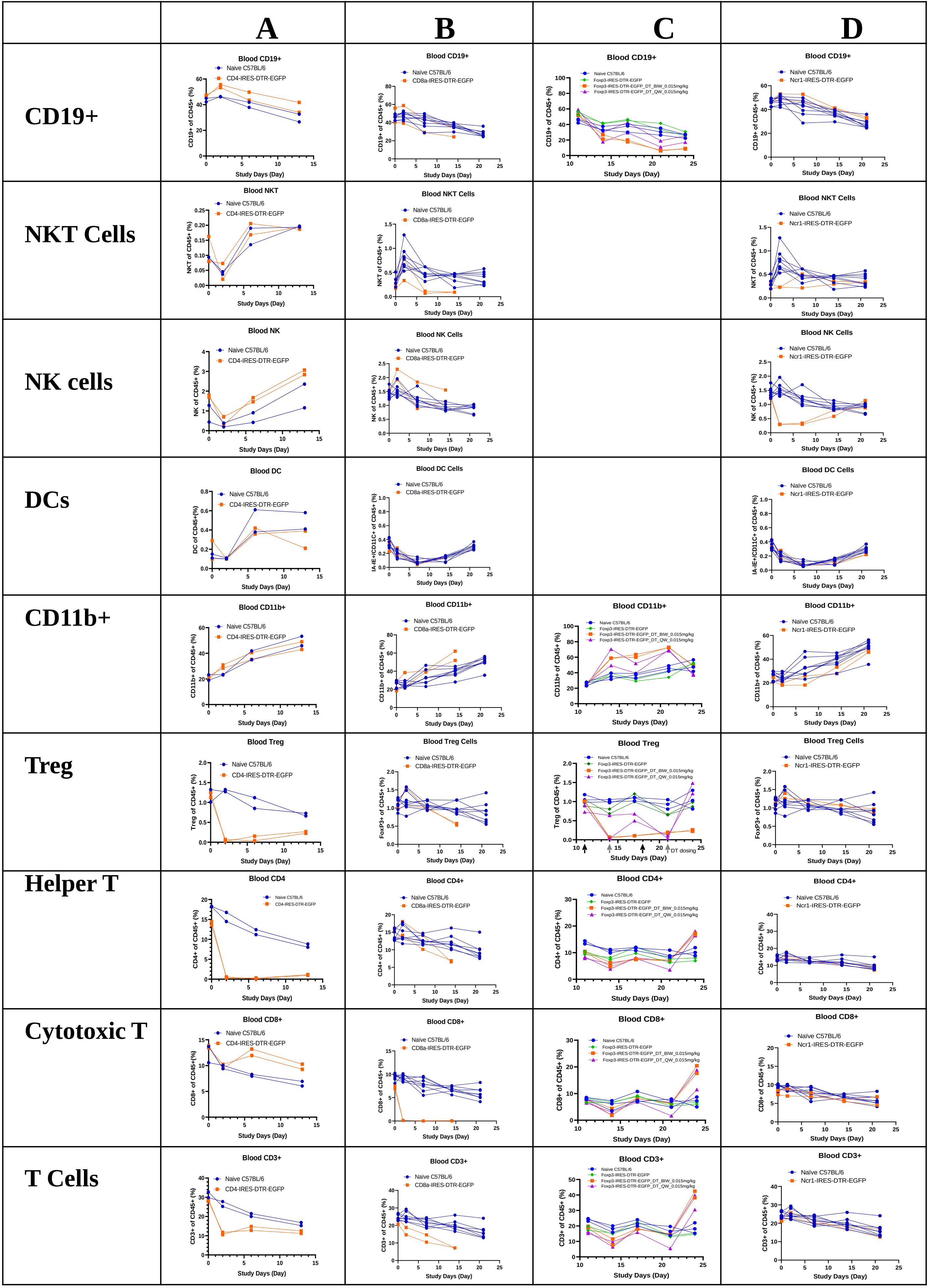

Supplement Figure 4; FACs assessment of individual immunes cells (%) in the blood in (column A) CD4-DTR mice (column B) CD8a-DTR mice (column C) Foxp3-DTR mice and (column D) Ncr1-DTR mice following treatment with DT (orange) in comparison to naïve WT mice (blue)

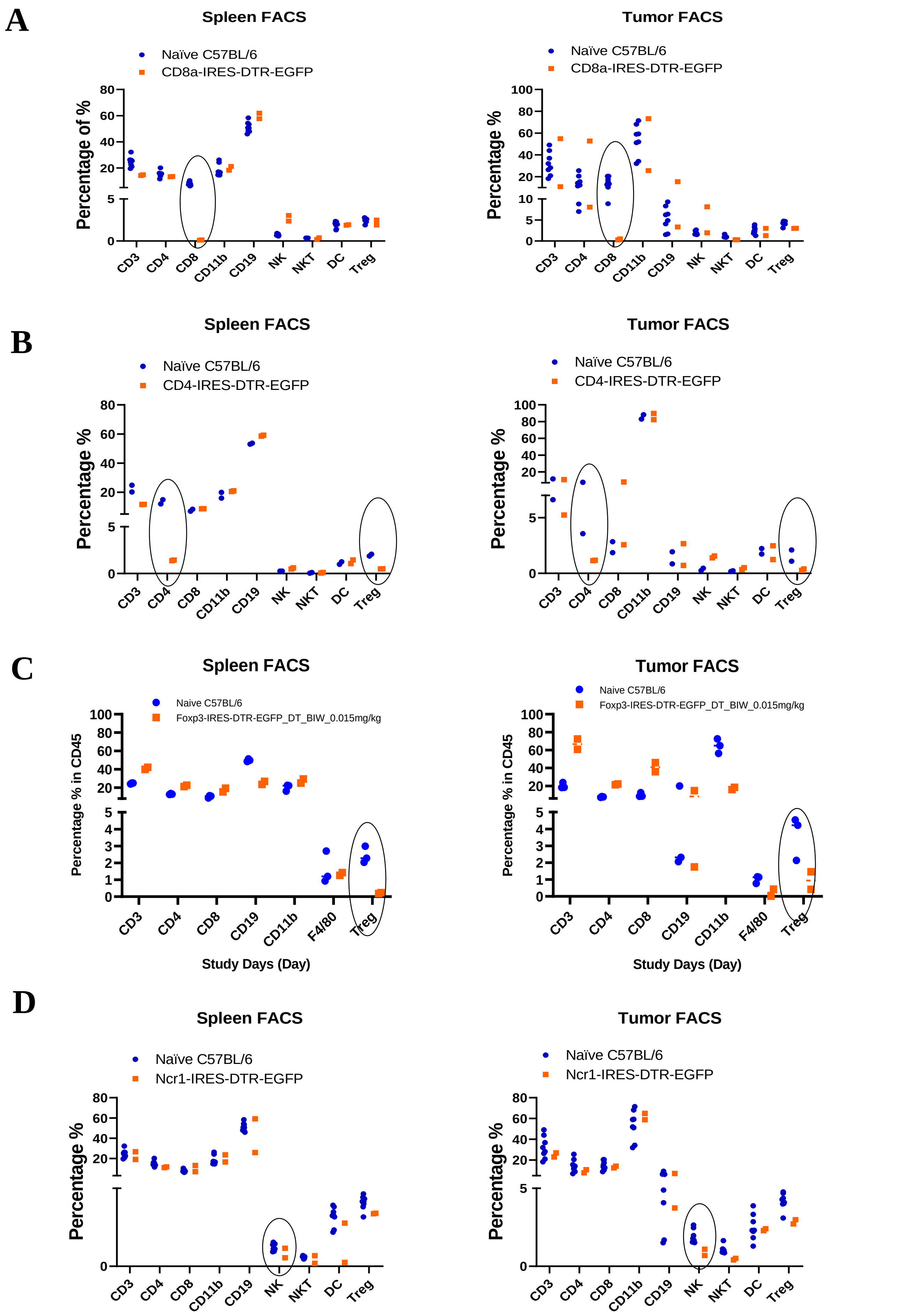

Supplement Figure 5: FACs assessment of immunes cells (%) in the spleen (left) and MC38 tumor (right) in A) CD8a-DTR mice B) CD4-DTR mice C) Foxp3-DTR mice and D) Ncr1-DTR mice following treatment with DT (orange) in comparison to naïve WT mice (blue)

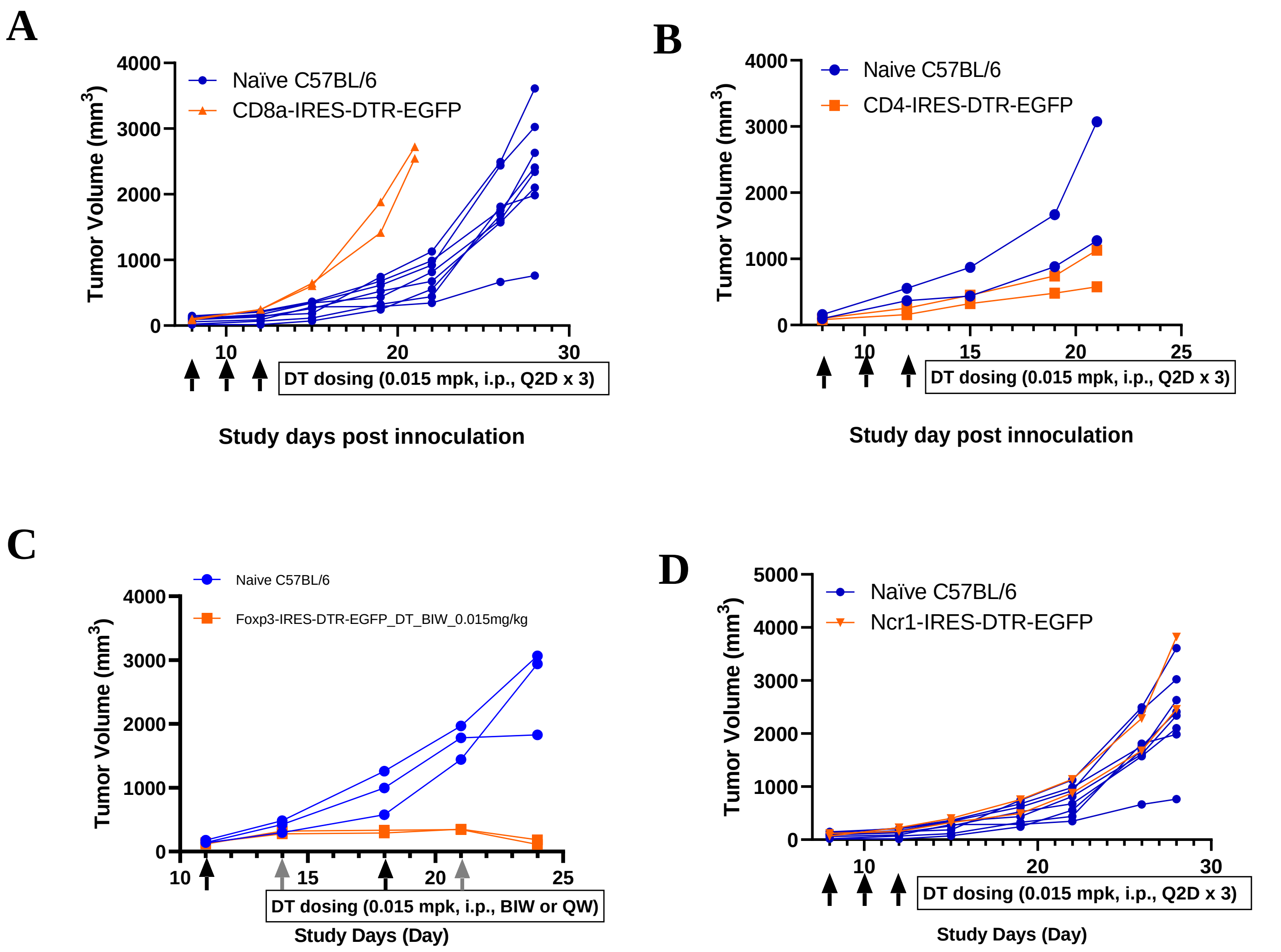

Supplemental Figure 6 Impact of targeted immune cell depletion on MC38 tumor growth in DTR transgenic mice A) CD8a-DTR mice, B CD4-DTR mice, C) Foxp3-DTR mice and D) Ncr1-DTR mice.

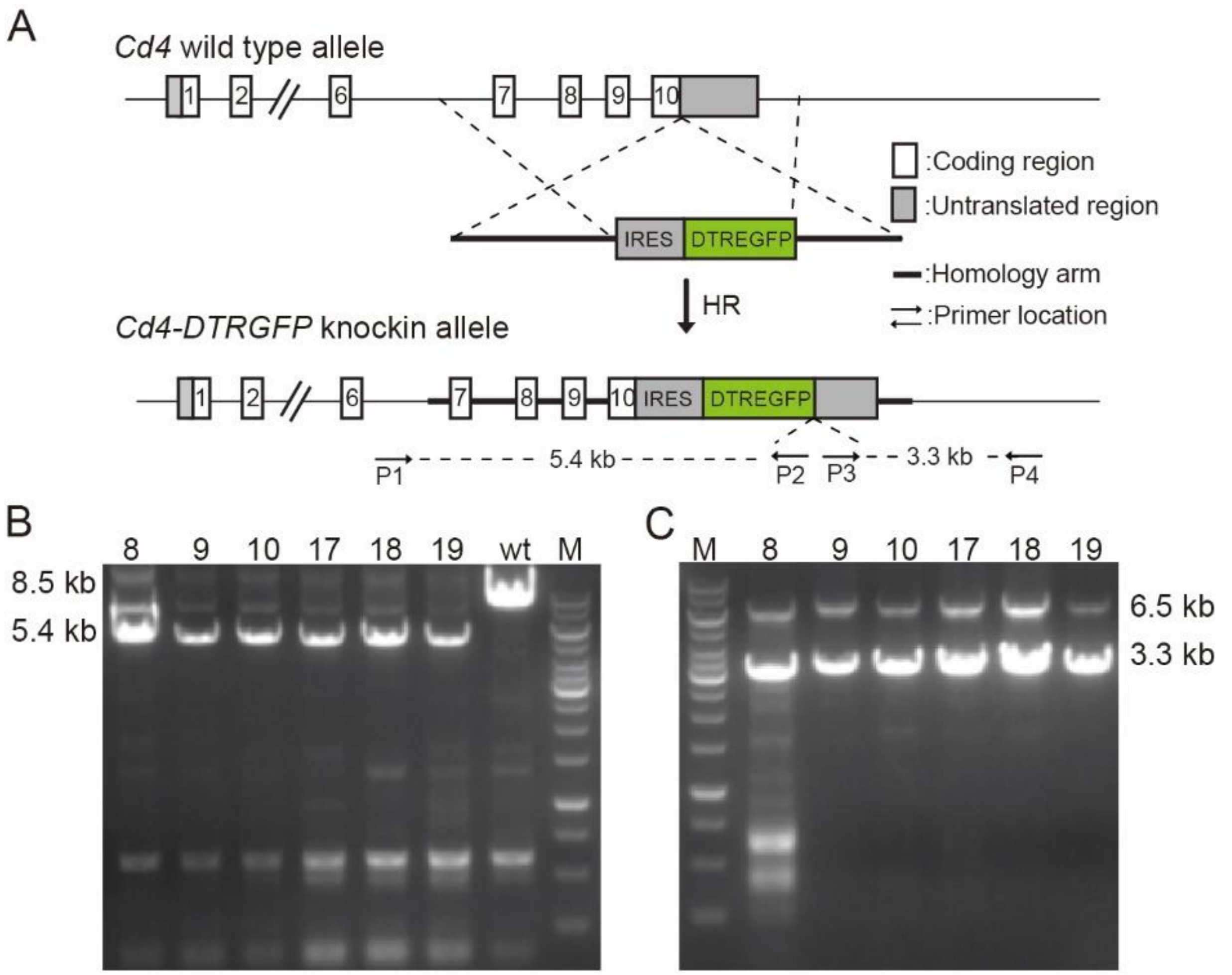

Supplemental Figure 7 Generation of Cd4-DTRGFP mouse model

Cd4-DTRGFP mice were generated by Shanghai Model Organisms Center, Inc (Shanghai, China). This model was generated by CRISPR/Cas9 system in C57BL/6 mouse background. A donor vector containing IRES-DTREGFP and two 3 kb homology arm were constructed. The sgRNA target site was 5'- CATAATCTCATCTGAGGCCT-3'. The donor vector with gRNA and Cas9 mRNA was microinjected into C57BL/6 fertilized eggs. F0 generation mice positive for homologous recombination were identified by long PCR. The primers (P1-P4) used for genotyping the correct homology recombination were P1: 5'-GGCCAGACAAGACCTCCCAGTTAG -3' and P2: 5'- GCATAGTAGCGCTCCCCGTTGTTG -3' for the correct 5' homology arm recombination, and P3: 5'-AGCCGCCAAGAGTCCAGGTTAGTT-3' and P4: 5'- GGAGGGTTCTGGTCTTAGTGATAC-3' for the correct 3' homology arm recombination. The PCR products were further confirmed by sequencing. The F0 positive mice mate with C57BL/6 to get the F1 heterozygous mice. The genotype of F1 generation Cd4-DTRGFP mice were identified by long PCR and the PCR condition were same with genotype of F0 mice. The F1 mice were bred to obtain the Cd4-DTRGFP mice used in the experiments.

A) Schematic representation of the recombination strategy for Cd4-DTRGFP mouse model.

B-C) The representation of PCR identifying of Cd4 positive F1 generation mice using primers P1-P4 (primer location was indicated in A). Primer sets P1 and P2, which identified the recombination of 5'-homology arm, can amplify two bands of 8.5 and 5.4 kb in positive mice, and only 8.5 kb in wild-type mice (B). Primer sets P3 and P4, which identified the recombination of 3'-homology arm, can amplify two bands of 6.5 and 3.3 kb in positive mice, and only 6.5 kb in wild-type mice (C). (The number above the Figure B and C is the mouse number; 8,9,10,17,18,19 : positive F1 generation mice; wt: wild type control; M: Fermentas 1kb DNA ladder).

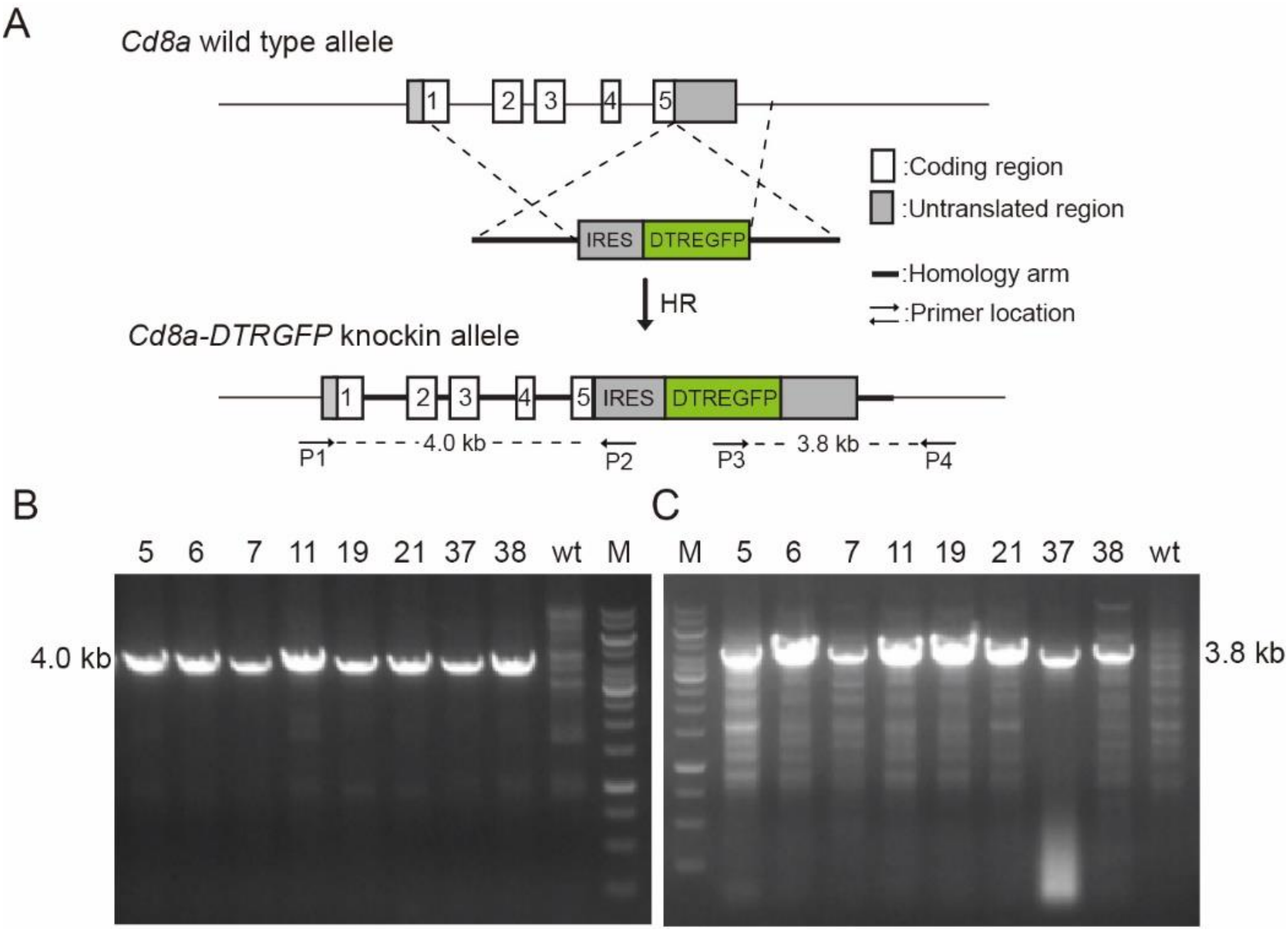

Supplemental Figure 8 Generation of Cd8a-DTRGFP mouse model

Cd8a-DTRGFP mice were generated by Shanghai Model Organisms Center, Inc (Shanghai, China). This model was generated by CRISPR/Cas9 system in C57BL/6 mouse background. A donor vector containing IRES-DTREGFP and two 3 kb homology arm were constructed. The sgRNA target site was 5'- TCAGAGAAAATTGTGTAAAA -3'. The donor vector with gRNA and Cas9 mRNA was microinjected into C57BL/6 fertilized eggs. F0 generation mice positive for homologous recombination were identified by long PCR. The primers (P1-P4) used for genotyping the correct homology recombination were P1: 5'- CGCACCTCCTCCGCCCTGTTCCT -3' and P2: 5'- TTGTGGCCATATTATCATCGTGTT -3' for the correct 5' homology arm recombination, and P3: 5'- CCCGCGCCGAGGTGAAG-3' and P4: 5'- AGTTAGCTGGTGGTGGTGGTTGC -3' for the correct 3' homology arm recombination. The PCR products were further confirmed by sequencing. The F0 positive mice mate with C57BL/6 to get the F1 heterozygous mice. The genotype of F1 generation Cd8a-DTRGFP mice were identified by long PCR and the PCR condition were same with genotype of F0 mice. The F1 mice were bred to obtain the Cd8a-DTRGFP mice used in the experiments.

A: Schematic representation of the recombination strategy for Cd8a-DTRGFP mouse model.

B-C: The representation of PCR identifying of Cd8a positive F0 generation mice using primers P1-P4 (primer location was indicated in A). Primer sets P1 and P2, which identified the recombination of 5'-homology arm, yielded one fragment (4.0 kb) in positive mice (B). Primer sets P3 and P4, which identified the recombination of 3'-homology arm, yielded one fragment (3.8 kb) in positive mice (C). (The number above the Figure B and C is the mouse number; wt: wild type control; M: Fermentas 1kb DNA ladder).

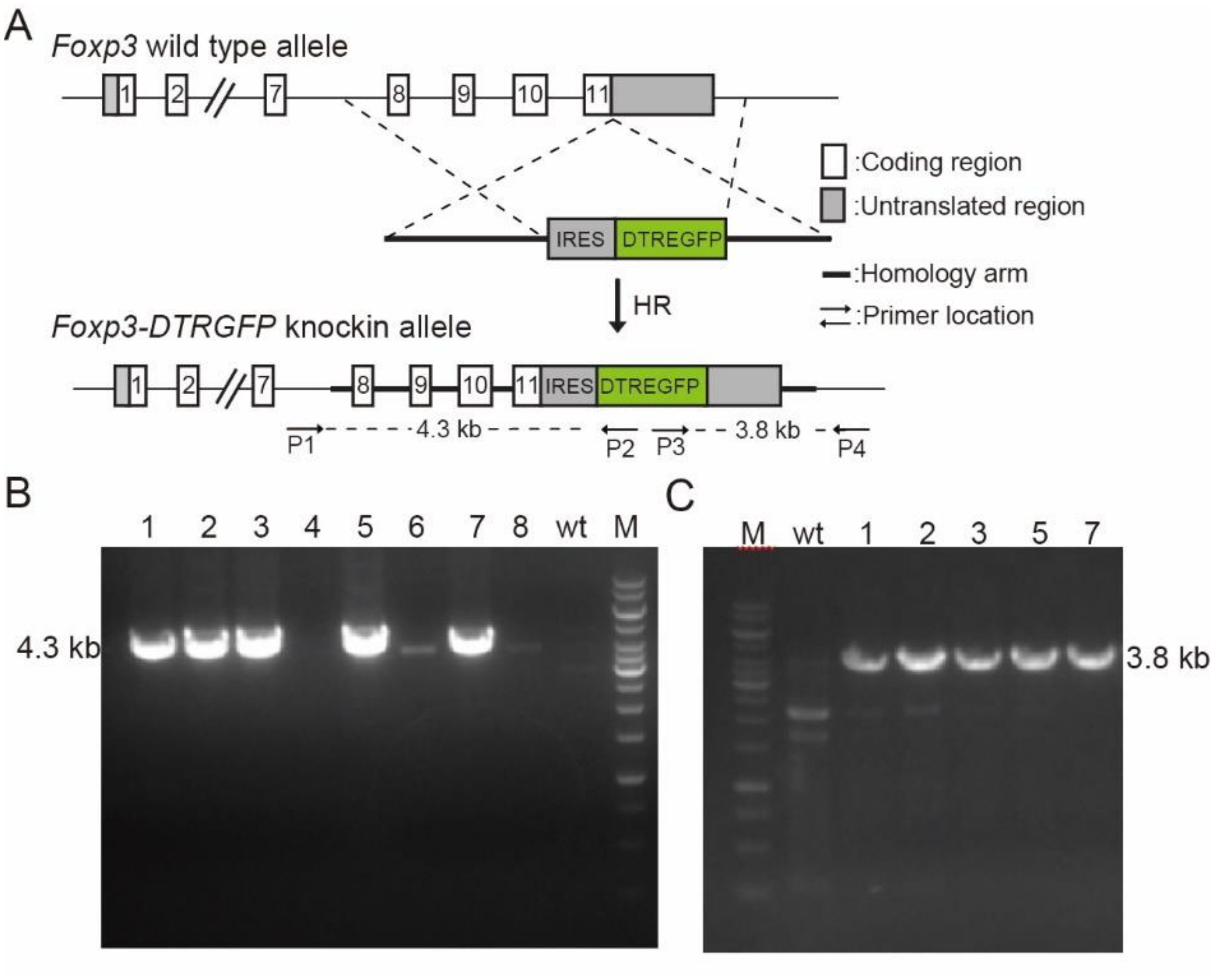

Supplemental Figure 9 Generation of Foxp3-DTRGFP mouse model

Foxp3-DTRGFP mice were generated by Shanghai Model Organisms Center, Inc (Shanghai, China). This model was generated by CRISPR/Cas9 system in C57BL/6 mouse background. A donor vector containing IRES-DTREGFP and two 3 kb homology arm were constructed. The sgRNA target sites were 5'-CAGGGATTGGAGCACTTGTT-3'. The donor vector with gRNA and Cas9 mRNA was microinjected into C57BL/6 fertilized eggs. F0 generation mice positive for homologous recombination were identified by long PCR. The primers (P1-P4) used for genotyping the correct homology recombination were P1: 5'-AAAATGGAGGGCCTTATGTTCA-3' and P2: 5'- AAGGGTCCGGGTTGCTGGTTC-3' for the correct 5' homology arm recombination, and P3: 5'- ATGGCCGACAAGCAGAAGAACG-3' and P4: 5'- GTGAGCAGCTGGCGAGTGTGAA-3' for the correct 3' homology arm recombination. The PCR products were further confirmed by sequencing. The F0 positive mice mate with C57BL/6 to get the F1 heterozygous mice. The genotype of F1 generation Foxp3-DTRGFP mice were identified by long PCR and the PCR condition were same with genotype of F0 mice.The F1 mice were bred to obtain the homozygous mice used in the experiments.

A) Schematic representation of the recombination strategy for Foxp3-DTRGFP mouse model.

B-C) The representation of PCR identifying of Foxp3 positive F1 generation mice using prmiers P1-P4 (primer location was indicated in A). Primer sets P1 and P2, which identified the recombination of 5'-homology arm, yielded one fragment (4.3 kb) in positive mice (B). Primer sets P3 and P4, which identified the recombination of 3'-homology arm, yielded one fragment (3.8 kb) in positive mice (C). (wt: wild type control; 1,2,3,5,7: positive F1 generation mice; M: Fermentas 1kb DNA ladder).

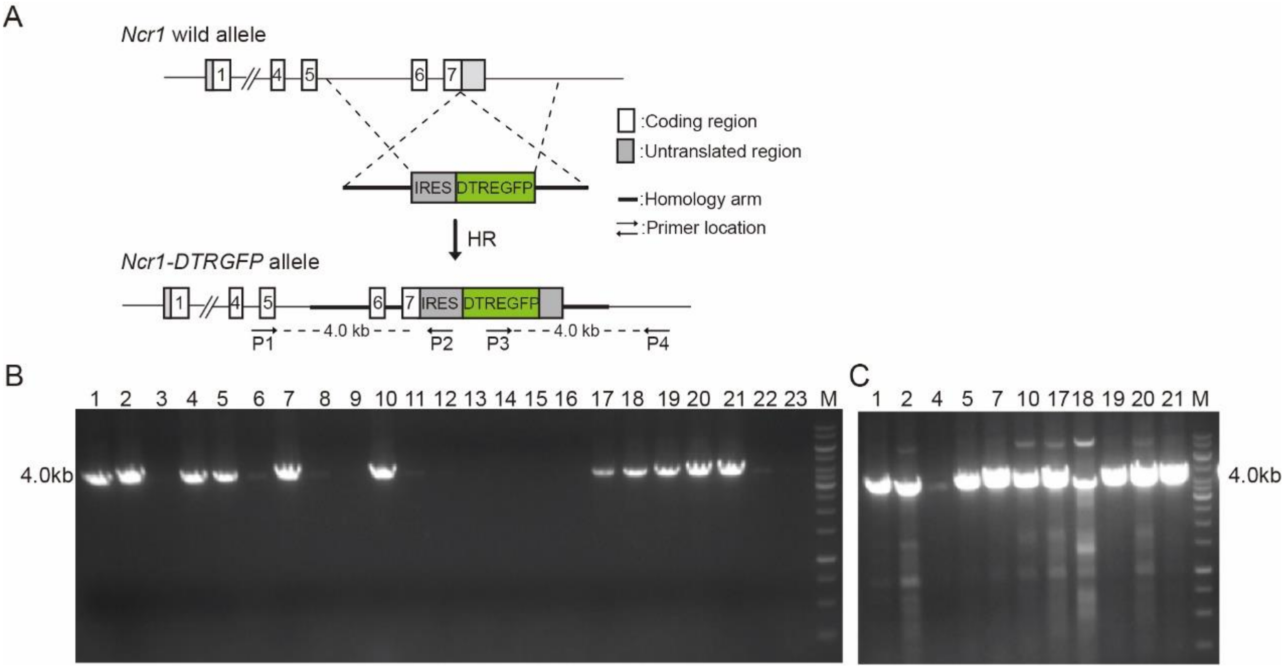

Supplemental Figure 10 Generation of Ncr1-DTRGFP mouse model

Ncr1-DTRGFP mice were generated by Shanghai Model Organisms Center, Inc (Shanghai, China). This model was generated by CRISPR/Cas9 system in C57BL/6 mouse background. A donor vector containing IRES-DTREGFP and two 3 kb homology arm were constructed. The sgRNA target site was 5'- GGTACAGCATAGAGCTCACA -3'. The donor vector with gRNA and Cas9 mRNA was microinjected into C57BL/6 fertilized eggs. F0 generation mice positive for homologous recombination were identified by long PCR. The primers (P1-P4) used for genotyping the correct homology recombination were P1: 5'-AGCCTTGCACCTACCGACCCTACT-3' and P2: 5'- TGTGGCCATATTATCATCGTGTTT-3' for the correct 5' homology arm recombination, and P3: 5'- CAGCCGCTACCCCGACCACA-3' and P4: 5'- CCCCCTCTTGCCTTCTTACTCC-3' for the correct 3' homology arm recombination. The PCR products were further confirmed by sequencing. The F0 positive mice mate with C57BL/6 to get the F1 heterozygous mice. The genotype of F1 generation Ncr1-DTRGFP mice were identified by long PCR and the PCR condition were same with genotype of F0 mice. The F1 mice were bred to obtain the Ncr1-DTRGFP mice used in the experiments..

A: Schematic representation of the recombination strategy for Ncr1-DTRGFP mouse model.

B-C: The representation of PCR identifying of Ncr1 positive F0 generation mice using primers P1-P4 (primer location was indicated in A). Primer sets P1 and P2, which identified the recombination of 5'-homology arm, yielded one fragment (4.0 kb) in positive mice (B). Primer sets P3 and P4, which identified the recombination of 3'-homology arm, yielded one fragment (4.0 kb) in positive mice (C). (The number above the Figure B and C is the mouse number; M: Fermentas 1kb DNA ladder).
